# Selective bromochlorination of a homoallylic alcohol for the total synthesis of (−)-anverene

**DOI:** 10.3762/bjoc.12.129

**Published:** 2016-07-01

**Authors:** Frederick J Seidl, Noah Z Burns

**Affiliations:** 1 Department of Chemistry, Stanford University, Stanford, California 94305, USA

**Keywords:** enantioselective catalysis, halogenation, natural products, total synthesis

## Abstract

The scope of a recently reported method for the catalytic enantioselective bromochlorination of allylic alcohols is expanded to include a specific homoallylic alcohol. Critical factors for optimization of this reaction are highlighted. The utility of the product bromochloride is demonstrated by the first total synthesis of an antibacterial polyhalogenated monoterpene, (−)-anverene.

## Introduction

The directed enantioselective functionalization of olefins is an extremely powerful tool in synthesis. A preeminent example is the Sharpless asymmetric epoxidation (SAE), which has been featured in countless syntheses of enantioenriched small molecules [1–2]. While the generality and scope of such catalytic enantioselective methods may be large, limitations often exist. In the case of the SAE, allylic alcohols are viable substrates, but homoallylic alcohols undergo epoxidation at slower rates and with diminished yields and enantioselectivities [3]. This particular limitation has inspired the development of several remote epoxidation methods for homoallylic and bishomoallylic alcohols [4–5], now allowing chemists to directly access a greater variety of enantioenriched epoxides.

**Scheme 1:**
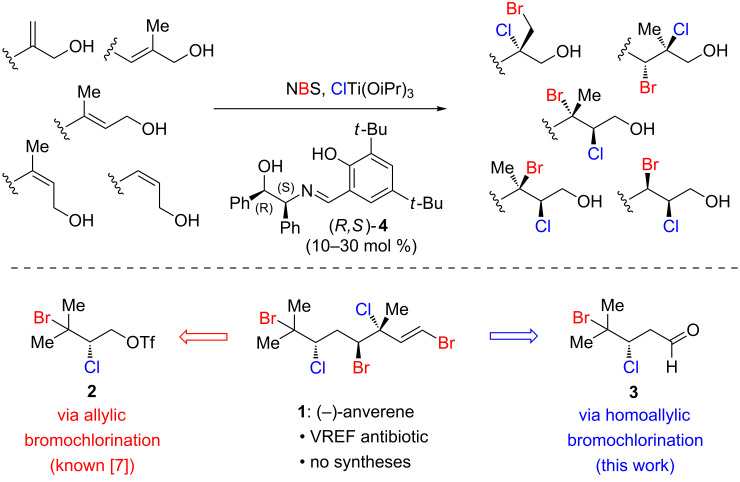
Selective bromochlorination and possible disconnections for anverene (**1**).

Several years ago our laboratory developed the first catalytic enantioselective dibromination of cinnamyl alcohols [6]. Unfortunately, this method suffered from the requirement for electronically biased substrates, and the catalyst system was not applicable to other dihalogenation reactions. In 2015, we disclosed a much-improved catalytic enantioselective bromochlorination protocol that is able to override inherent substrate regioselectivity [7]. This development vastly expanded the substrate scope to include a larger subset of alkyl-substituted allylic alcohols (Scheme 1, top); the relative and absolute stereochemical configurations of all resulting bromochlorides have been assigned and are shown for the (*R*,*S*) Schiff base ligand [7–9]. Furthermore, the reaction conditions could now be modulated to affect both catalytic enantioselective dichlorination and dibromination of allylic alcohols [9]. The utility of this method has been demonstrated by highly selective total syntheses of several important polyhalogenated natural product targets including (+)-halomon [8] and (−)-danicalipin A [9].

While exploring the scope of this bromochlorination reaction, we discovered that slight modifications allow for the bromochlorination of a homoallylic alcohol with synthetically useful levels of regio- and enantioselectivity. Disclosed herein, this discovery has enabled the first total synthesis of (−)-anverene (**1**) (Scheme 1, bottom), a secondary metabolite from the algae *Plocamium cartilagineum* with selective antibiotic activity against vancomycin-resistant *Enterococcus faecium* (VREF) [10]. (−)-Anverene (**1**) is a member of a large and synthetically challenging family of polyhalogenated acyclic monoterpenes, for which few stereoselective synthetic approaches exist [11].

## Results and Discussion

In considering a retrosynthesis of anverene (**1**) we initially identified prenol bromochlorotriflate **2** (Scheme 1, bottom) as a useful synthon, as it is readily accessible from prenol using our original bromochlorination protocol [7]. In a forward sense, this intermediate could be coupled with a vinyl organometallic to introduce the remainder of the carbon skeleton of **1**. While we have achieved moderate success in the copper-catalyzed cross coupling of an analogous dichlorotriflate [9], in our hands **2** failed to engage in productive carbon–carbon bond formation. An alternative retrosynthesis traced **1** back to bromochloroaldehyde **3**, which should readily engage in standard olefination reactions. As **3** could arise from a bromochlorinated homoallylic alcohol, we viewed this as an impetus for developing conditions for the bromochlorination of 4-methyl-3-pentenol (homoprenol, **5**).

### Optimization

Under our published conditions for allylic alcohols, homoprenol (**5**) underwent bromochlorination to deliver the target isomer **6** in 46% yield and 84% ee (Table 1, entry 1). Constitutional isomer **7** was also observed (4:1 ratio of **6**:**7**), as well as dibromide **8** and other racemic brominated byproducts (see Supporting Information File 1). The absolute configuration of **6** was assigned through its subsequent conversion into (−)-anverene (**1**, see below), the absolute configuration of which was determined by the isolation chemists on natural material by X-ray crystallography [10]. Interestingly, using the same enantiomer of ligand, the bromochlorides derived from prenol and homoprenol (**5**) have the same absolute configuration. This is in stark contrast to the SAE, for which the facial selectivity of epoxidation for homoallylic alcohols is the opposite of that seen for allylic alcohols [3]. Additional information on the scope of the bromochlorination for other homoallylic alcohols is presented in Supporting Information File 1. In the subsequent optimization, three critical factors came to the fore: concentration, the inclusion of additional Ti(OiPr)_4_, and stirring rate.

**Table 1:** Optimization of a selective homoallylic bromochlorination reaction.^a^

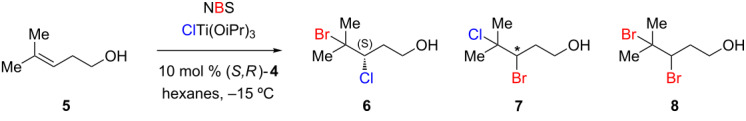						

entry	additive	concentration	yield **6**/ee **6** (%)	yield **7**/ee **7** (%)	cr (**6**:**7**)	yield **8**, others (%)

1	–	0.1 M	46/84	13/23	4:1	6, 34
2	–	0.025 M	45/82	10/19	5:1	3, 27
3	Ti(OiPr)_4_^b^	0.1 M	50/89	10/18	5:1	7, 28
4	Ti(OiPr)_4_^b^	0.025 M	57/89	6/38	10:1	6, 16

^a^Reactions were conducted on 0.1 mmol scale with 1.2 equiv NBS, 1.1 equiv ClTi(OiPr)_3_; ^b^20 mol %.

When unoptimized bromochlorination reactions of homoprenol were quenched at low and high conversion, different distributions of side products were obtained. Unlike Table 1, entry 1, which was taken to completion, bromochlorinations quenched at low conversion exhibited high enantioselectivity for **6**, but also constitutional isomer ratios (cr) as low as 2:1 for **6**:**7** (not shown). These observations are consistent with a highly selective catalytic reaction competing with several nonselective background processes. By both lowering the concentration and adding 20 mol % of Ti(OiPr)_4_ (Table 1, entries 2–4), much of the background reactivity was minimized; adding more than 20 mol % of Ti(OiPr)_4_ did not result in further improvement. While the structure of the active bromochlorination catalyst is not yet known, we speculate that the procedural changes in entries 2–4 do not alter its structure, but rather change the distribution of titanium species responsible for off-cycle reactivity. Titanium alkoxides are known to undergo facile ligand exchange at −20 °C, and can adopt both monomeric and oligomeric structures, several of which can be catalytically active [12]. When Cl_2_Ti(OiPr)_2_ is used in place of ClTi(OiPr)_3_, bromochloride **7** forms very quickly with no enantioselectivity and with inherent substrate regioselectivity. The additional Ti(OiPr)_4_ may limit the trace formation of these highly reactive dichlorotitanium species early in the reaction, thereby preventing background bromochlorination. Lowering the concentration of the reaction may favour lower-order titanium aggregates over oligomeric species [12].

It has been our observation that high stirring rates are critical for obtaining optimal results in these heterogeneous bromochlorination reactions of both allylic and homoallylic alcohols. The origin of this stirring dependence is not definitively known, but we suspect that it facilitates the dissolution of *N*-bromosuccinimide (NBS) into hexanes and/or its incorporation into the active catalyst. The bromochlorination of homoprenol (**5**) proved to be especially sensitive to stirring. This may be due to the slower rate of selective bromochlorination of homoallylic alcohols, leading to closer competition with nonselective background halogenation than the same reaction with allylic alcohols. Consistent results were obtained when bromochlorination reactions were run in round-bottomed flasks, using rod-shaped magnetic stir bars roughly the length of the flask radius, and with stirring at no less than 1500 rpm (see Supporting Information File 1 for images). Under optimized conditions, bromochloroalcohol **6** was produced in 57% yield and 89% enantiomeric excess (ee) with small amounts of undesired bromochloride **7** (6%) and other brominated byproducts (**8** + others, 22% combined) (Table 1, entry 4). The trace dibromide **8** was quantitatively and chemoselectively decomposed, via a reductive de-dihalogenation pathway to the corresponding olefin, by heating the crude material to reflux in acetone with sodium iodide.

### Total synthesis of anverene

**Scheme 2:**
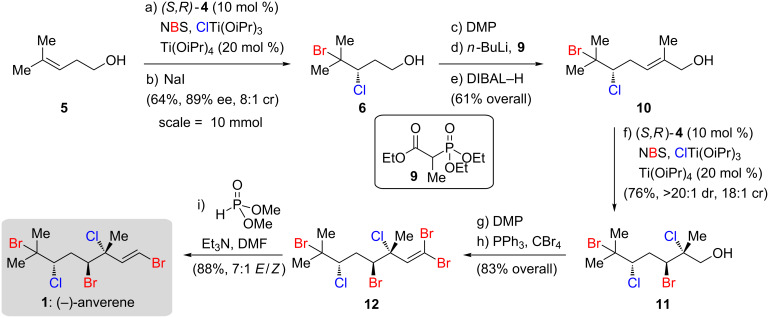
Selective total synthesis of (−)-anverene. Reagents and conditions: a) NBS (1.2 equiv), ClTi(OiPr)_3_ (1.1 equiv), 20 mol % Ti(OiPr)_4_, 10 mol % (*S,R*)-**4**, 0.025 M hexanes, −15 °C, 17 h; b) NaI (2 equiv), acetone, 65 °C, 3 h; c) Dess–Martin periodinane (1.2 equiv), NaHCO_3_ (5 equiv), CH_2_Cl_2_, 0 °C to rt; d) triethyl 2-phosphonopropionate **9** (1.5 equiv), *n*-BuLi (1.2 equiv), 1:1 THF/acetonitrile, 0 °C, 5 min; e) DIBAL−H (3 equiv), CH_2_Cl_2_, −78 °C, 20 min; f*)* NBS (1.2 equiv), ClTi(OiPr)_3_ (1.1 equiv), 20 mol % Ti(OiPr)_4_, 10 mol % (*S,R*)-**4**, 0.025 M hexanes, −15 °C, 42 h; g) Dess–Martin periodinane (1.2 equiv), NaHCO_3_ (5 equiv), CH_2_Cl_2_, 0 °C to rt; h) CBr_4_ (2 equiv), PPh_3_ (4 equiv), CH_2_Cl_2_, 0 °C, 10 min; i) Et_3_N (5 equiv), dimethyl phosphonate (4 equiv), DMF, rt, 5 min.

With scalable access to **6**, a total synthesis of (−)-anverene (**1**) was explored (Scheme 2). Homoprenol (**5**) was subjected to the two-step bromochlorination/de-dibromination protocol on 10 mmol scale, furnishing bromochloride **6** in 64% overall yield and 89% ee as an 8:1 ratio of constitutionally isomeric bromochlorides. Early incorporation of the sensitive vicinal bromochloride moiety in **6** necessitated a strategy that avoided exposure to strong bases or reducing agents, a limitation that significantly guided the choice of reagents and conditions for the subsequent steps. Dess–Martin periodinane oxidation of alcohol **6** followed by Horner–Wadsworth–Emmons olefination with triethyl 2-phosphonopropionate (**9**) furnished the targeted unsaturated ester (not shown). In initial investigations into the formation of this unsaturated ester, Masamune–Roush conditions [13] were attempted but led to sluggish reactivity and concomitant base-mediated elimination of the bromochloride. This decomposition pathway was minimized by the stoichiometric generation of the lithium carbanion of **9** with *n*-BuLi prior to addition of the corresponding aldehyde, and by maintaining very short (<10 minutes) reaction times at 0 °C. This protocol led to complete conversion exclusively to the *E*-unsaturated ester without any observed elimination.

The unsaturated ester was then reduced with DiBAl–H to allylic alcohol **10**, which was isolated as an unchanged 8:1 mixture of regioisomeric bromochlorides, strongly suggesting that no stereochemical or regiochemical isomerization had taken place over the previous three steps from **6**. When **10** was subjected to ligandless bromochlorination with ClTi(OiPr)_3_ and NBS in hexanes, a surprisingly high dr of 7:1 favouring tetrahalide **11** was observed, indicating significant inherent substrate diastereocontrol for the *anti*-1,3-dihalide over the *syn* alternative. Alternatively, when **10** was subjected to the bromochlorination protocol with 10 mol % (*S,R*)-**4**, the product tetrahalide **11** was isolated in >20:1 dr favouring the same *anti*-1,3-dihalide. Interestingly, we observed considerable enrichment in the regioisomeric bromochloride ratio for the left hand side, which had roughly doubled from 8:1 to 18:1, and the recovered starting material was found to have a regioisomeric bromochloride ratio that was lowered to 1.6:1. Taking into account that the minor bromochloride constitutional isomer (**7**, Table 1) is formed in 20–40% ee, these results are consistent with the catalyst system being capable of selective resolution between two enantiomers (or pseudoenantiomers) of a substrate allylic alcohol.

Tetrahalide **11** was then oxidized to the corresponding aldehyde. Installation of the vinyl bromide was found to be difficult using traditional methods. Takai olefination [14] with CHBr_3_ and CrBr_2_ resulted in significant de-dihalogenation, likely due to the presence of reducing agents such as Cr(II) or residual LiAlH_4_ from the preparation of CrBr_2_. Fortuitously, an efficient and high-yielding alternative to these routes was found. Following Ramirez dibromomethylenation [15], 1,1-dibromoolefin **12** was isolated and subsequently reduced with dimethyl phosphonate [16–17]. While the mechanism of this Hirao reduction is not fully understood, the reaction was exquisitely mild and proceeded with high chemoselectivity for the geminal dihalide, delivering (−)-anverene (**1**) in high yield as a 7:1 mixture of *E*/*Z* isomers. Following recrystallization, over 100 mg of **1** was isolated as a single diastereomer and constitutional isomer, culminating a 9-step route with an overall yield of 21.8%.

## Conclusion

In conclusion, a new protocol for the catalytic enantioselective bromochlorination reaction has been developed which has enabled the highly efficient and selective synthesis of **1** via homoprenol bromochloride **6**. The factors addressed in the optimization of the bromochlorination for homoprenol, namely concentration, the inclusion of additional Ti(OiPr)_4_, and vigorous stirring, are expected to be generally important to other challenging substrates. Work is ongoing in our laboratory to better understand and predict the matched/mismatched effect seen for chiral substrates, in particular by studying the kinetic resolution of racemic allylic alcohols. Detailed mechanistic and computational studies are underway to better understand the active catalyst structure and the origins of selectivity.

## Supporting Information

File 1 Experimental procedures, full characterization of new compounds, and spectral data.
