# Supplementary material for: Selective bromochlorination of a homoallylic alcohol for the total synthesis of (−)-anverene
Source: Beilstein J Org Chem. 2016 Jul 1;12:1361–5. doi: 10.3762/bjoc.12.129 (PMC4979643; doi:10.3762/bjoc.12.129)
Supplement: File 1 — Experimental procedures, full characterization of new compounds, and spectral data. [file Beilstein_J_Org_Chem-12-1361-s001.pdf]

Supporting Information

for

Selective bromochlorination of a homoallylic alcohol for the total synthesis of (−)-anverene

Frederick J. Seidl and Noah Z. Burns*

Address: Department of Chemistry, Stanford University, Stanford, California 94305, USA

Email: Noah Z. Burns - nburns@stanford.edu

* Corresponding author

Experimental procedures, full characterization of new compounds, and spectral data

1. General information S2
2. Additional information on reaction scope S3
3. Preparation and characterization of products S5
4. ^{1}H and ^{13}C NMR spectra S15
5. Comparison of synthetic and natural **1** S30
6. HPLC traces of racemic and scalemic **6** S32
7. References S33

1. General information

All reactions were conducted in oven- or flame-dried glassware under an atmosphere of nitrogen or argon unless otherwise noted. Commercial reagents and solvents were used as received unless otherwise noted with the exception of the following: hexanes (ACS grade, 4.2% various methylpentanes), toluene, tetrahydrofuran, acetonitrile, methanol, benzene, and dichloromethane were dried by passing through a bed of activated alumina in a JC Meyer Solvent System. Flash column chromatography was performed using F60 silica gel (40–63 μm, 230–400 mesh, 60 Å) purchased from Silicycle. Analytical thin-layer chromatography (TLC) was carried out on 250 μm 60-F_{254} silica gel plates purchased from EMD Millipore, and visualization was effected by observation of fluorescence-quenching with ultraviolet light and staining with either *p*-anisaldehyde. Proton nuclear magnetic resonance (^{1}H NMR) and carbon nuclear magnetic resonance (^{13}C NMR) spectra were recorded on Varian Inova 600 or Varian Inova 500 spectrometers operating at 600 and 500 MHz respectively for ^{1}H, and at 150 and 125 MHz for ^{13}C. Chemical shifts are reported in parts per million (ppm) with respect to residual protonated solvent for ^{1}H ($CHCl_3$ = δ 7.26) and with respect to carbon resonances of the solvent for ^{13}C ($CDCl_3$ = δ 77.0). Peak multiplicities are reported as follows: app = apparent, br = broad, s = singlet, d = doublet, t = triplet, q = quartet, m = multiplet. ^{1}H NMR yields for small-scale reactions were determined by comparison to 1,3,5-trimethoxybenzene as an internal standard. Infrared (IR) spectra were recorded on a Nicolet 6700 FT-IR spectrometer. GC–MS (CI) data were collected on a Waters Micromass GCT Premier mass spectrometer. Optical rotations were measured using a JASCO P-2000 polarimeter. Chiral high-performance liquid chromatography (HPLC) analysis was performed using an Agilent 1260 with commercial ChiralCel or ChiralPak 4.6 × 250 mm columns. HPLC trace integration was performed automatically by the Agilent 1260's accompanying OpenLab processing suite. Uncorrected melting point data were collected using a Thomas Hoover Uni-Melt apparatus.

2. Additional information on reaction scope

Table S1: Scope of bromochlorination for selected substrates:

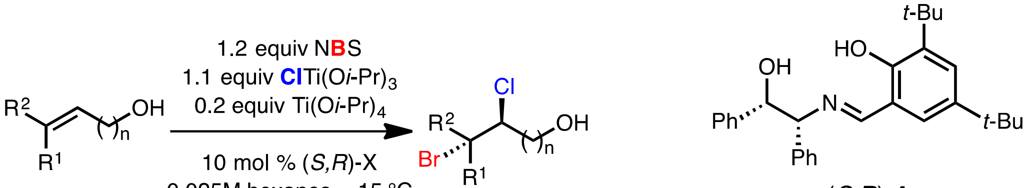

(*S*,*R*)-**4**

Entry	Substrate	Product(s)	yield (%)	ee (%)	cr	BrCl:Br_2
	Allylic Alcohols:					
**1**	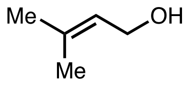 **S1a**	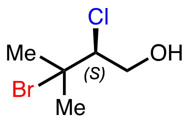 **S1b**	82	96	7:1	>20:1
**2**	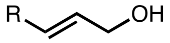 **S2a** (R = n-C_5H_{11})	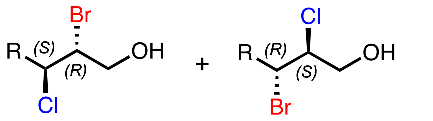 **S2b** (major, 98% ee) **S2c** (minor, 50% ee)	81	--	~2:1	11:1
**3**	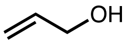 **S3a**	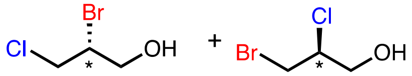 **S3b** (major, 91% ee) **S3** (minor, 30% ee)	10-30	--	~1:1	10:1
	Homoallylic Alcohols:					
**4**	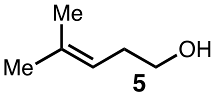 **5**	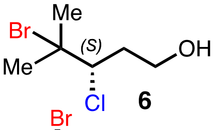 **6**	64	89	8:1	10:1
**5**	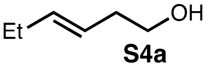 **S4a**	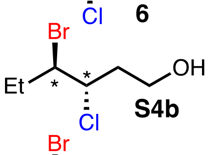 **S4b**	30-50	~80[a]	10:1	5:1
**6**	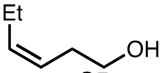 **S5a**	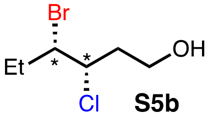 **S5b**	30-50	~80[a]	14:1	4:1
**7**	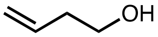 **S6a**	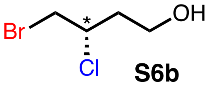 **S6b**	10-30	~80[a]	>20:1	~2:1

Substrates bromochlorinated with low/no enantioselectivity:

Allylic, cis-proximal

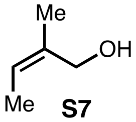 **S7**

Homoallylic, proximal substitution:

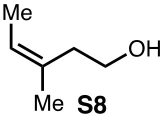 **S8**

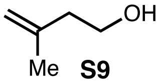 **S9**

Electronically biased homoallylic alcohols:

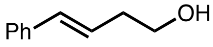 **S10**

Bishomoallylic alcohols:

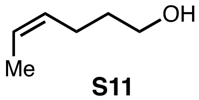 **S11**

[a]Low estimate of ee due to moderate peak overlap with dibromide in HPLC

Note: an asterisk (*) indicates that the absolute configuration has been assigned by analogy to other substrates. See the following text for details.

Discussion on the scope and mnemonic of the bromochlorination reaction:

Despite the clear utility of the bromochlorination reaction, it is not general to all allylic or homoallylic alcohols. The most capricious aspect of the bromochlorination reaction tends to be the regioselectivity, but there are also substrate classes for which no enantioselectivity is seen at all. These limitations are summarized here and in Table S1 above. The bromochlorination of prenol **S1a** is included for reference (Table S1, entry 1) [1]. Alkyl-*trans*-1,2-disubstituted allylic alcohols **s2a** and parent allyl alcohol **S3a** were bromochlorinated with high enantioselectivity, but with poor regioselectivity, rendering the resultant motifs completely inaccessible in pure form (Table S1, entries 2 and 3). Surprisingly, the major regioisomeric bromochloride arose from 6-*endo* chloride delivery to the distal olefinic carbon producing **S2b** and **S3b** in high enantioselectivity. However, the minor 5-*exo* regioisomeric bromochlorides **S2c** and **S3c** were formed with low enantioselectivity, favoring the opposite pseudoenantiomeric dihalide. This was proven by subjecting the inseparable mix of regioisomeric bromochlorides **S2b** and **S2c** to radical debromination conditions. The resulting known alkyl chlorides were chromatographically separable, permitting purification and assignment by optical rotation [2]. These results indicate that both regioisomeric bromochlorides arise from the same intermediate bromonium for this substrate, and the disparity in enantioselectivity among them suggests that most background reactivity is funneled into the 5-*exo* regioisomeric bromochloride **S2c**.

Homoprenol **6** (Table S1, entry 4) is not the only homoallylic alcohol that can be bromochlorinated with high enantioselectivity. *Cis*- or *trans*-1,2-disubstituted substrates **S4a** and **S5a**, as well as terminal olefin **S6a** were bromochlorinated with apparent high regio- and enantioselectivity (Table S1, entries 5–7). Unfortunately, the chemoselectivity for entries 5–7 was poorer than that seen for homoprenol (**5**); bromochlorides **S4b**, **S5b** and **S6b** were each contaminated with a significant amount of dibromide. De-dibromination of these dihalides with NaI was unsuccessful; the dibromide proved to be too robust, and no reactivity was seen after 3 hours. Further, low isolated yields were obtained for these bromochlorides due to their volatility. The bromochlorination of these substrates was not optimized any further, but the high regioselectivity seen for **S4b** and **S6b** indicates a potentially attractive alternative route to the inaccessible dihalide moieties in **S2c** and **S3c**, respectively.

Further examination into the scope of the bromochlorination reaction revealed some interesting trends with regard to the substitution pattern for both allylic and homoallylic alcohols. For example, the allylic alcohol **S7** is unique among all other allylic alcohols tried: it was bromochlorinated in under 10% ee. With regards to homoallylic alcohols, substitution at the proximal carbon, as seen in **S8** and **S9**, also resulted in no enantioselectivity. Homocinnamyl alcohol (**S10**), an electronically biased substrate, was bromochlorinated with high regioselectivity to yield the 7-*endo* bromochloride corresponding to substrate-controlled Markovnikov regioselectivity, but in only 20% ee, and with very poor chemoselectivity. Bromochlorination was attempted on one bishomoallylic alcohol, **S11**, but no enantioselectivity was seen for this substrate.

3. Preparation and characterization of products

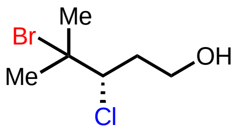

(−)-(*S*)-4-Bromo-3-chloro-4-methylpentan-1-ol (**6**): The (*S*)-enantiomer of **6** was prepared from homoprenol (**5**) [3] using the (*S,R*)-**4** ligand according to the following optimized bromochlorination procedure on 10 mmol scale. Dry hexanes (400 mL) were added to a dried 500 mL round-bottomed flask containing a 37 mm rod-shaped stirbar under N_2 atmosphere. Solid $ClTi(OiPr)_3$ (2.63 mL, 11 mmol, 1.1 equiv) was melted at 60 °C and added as a neat, viscous liquid by syringe using a long, thick (20 gauge) metal needle (commercial solutions of $ClTi(OiPr)_3$ in hexanes deliver identical results). $Ti(OiPr)_4$ (0.59 mL, 2 mmol, 0.2 equiv) was added as a neat liquid. Homoprenol (**5**, 1.00 g, 10.0 mmol, 1 equiv) was added as a solution in 3 mL hexanes. With vigorous stirring, (*S,R*)-**4** (430 mg, 1.0 mmol, 0.1 equiv) was added dropwise over 1 minute as a solution in 10 mL hexanes. The pale yellow homogeneous solution was brought to −15 °C in a cryocool. The septum and N_2 balloon were removed, briefly exposing the reaction to ambient air. *N*-Bromosuccinimide (2.14 g, 12 mmol, 1.2 equiv) was quickly added, and the reaction was capped with a polyethylene yellow stopper. The reaction was stirred at 1500 rpm, maintaining a strong vortex for 17 hours, after which TLC analysis of the pale yellow cloudy suspension indicated reaction completion. The reaction was quenched with 50 mL 1 M aq. Na_2SO_3, and allowed to warm to room temperature. Then, 100 mL ether and 20 mL conc. HCl were added, and the mixture was stirred vigorously for 15 minutes. The now clear layers were separated, the aqueous layer was washed 2 times with ether, and the combined organic layers were washed with 50 mL saturated aq. $NaHCO_3$. The combined organic layers were dried over Na_2SO_4 and solvent was removed by rotary evaporation yielding the crude bromochloride **6** as an orange oil, 2.51 g. ^1H NMR analysis indicated an 8.2:1.0:2.6 mixture of desired **6**:constitutional isomer **7**:dibromide **8**. HPLC analysis indicated the desired product **6** was formed in 89% ee.

To selectively de-dibrominate the undesired dibromide sideproduct, the crude oil was dissolved in 10 mL acetone in a scintillation vial, and NaI (600 mg, 4 mmol, roughly 2 equiv relative to dibromide) was added, yielding a fully homogeneous orange solution. The vial was capped tightly and brought to 65 °C in a sand bath with vigorous stirring for 3 hours. The dark brown opaque mixture was cooled to room temperature. TLC analysis indicated a mixture of **6** and **5**. The reaction was quenched with 10 mL 1 M aq. Na_2SO_3, and partitioned between 50 mL ether and 50 mL water. The layers were separated and the organic layer was washed with brine. The organic layer was dried over $MgSO_4$, filtered, and the solvent was removed by rotary evaporation. Most residual homoprenol was removed by rotary evaporation at room temperature and at a vacuum of 2–5 torr for 30 minutes. The dark brown crude oil was purified by column chromatography (silica gel, 25% ethyl acetate/hexanes) to yield the product **6** (1.38 g, 6.4 mmol, 64% yield) as an 8.2:1 mixture of **6**:**7** without dibromide contamination. HPLC analysis indicated the desired product remained at 89% ee.

Continued from last page:

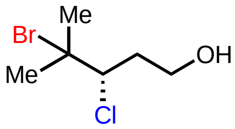

6

Physical properties: Viscous, pale orange oil; reversibly freezes upon refrigeration; m.p. not measured.

R*f* = 0.28 (silica gel, 25% ethyl acetate / hexanes, anisaldehyde stain);

IR (film) ν_{max} 3329 (br), 2975, 2933, 2887, 1456, 1423, 1387, 1371, 1226, 1103, 1055, 1036, 895, 853, 678, 669, 655, 589, 518 cm^{-1};

^{1}H NMR (600 MHz, $CDCl_3$): 4.25 (dd, 1H, *J* = 11.2, 1.8 Hz), 3.94-3.83 (m, 2H), 2.59 (dddd, 1H, *J* = 14.5, 9.3, 6.2, 1.8 Hz), 1.92 (s, 3H), 1.91-1.86 (m, 1H), 1.79 (s, 3H), 1.77 (br m, 1H);

^{13}C NMR (150 MHz, $CDCl_3$): 68.0 (C–Cl), 67.6, 59.9, 37.3, 33.3, 28.5;

GC/MS (CI): calcd. for $C_6H_{12}O^{79}Br^{35}Cl[NH_4]^+$: 232.0104, found 232.0111;

$[\alpha]_D^{23}$ = −37.0 (c = 1.0, $CHCl_3$) (at 88% ee).

Brominated sideproducts:

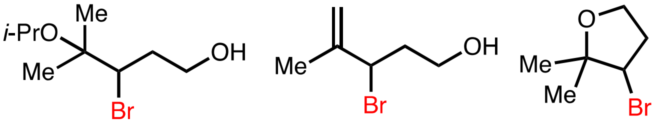

Figure S1: Reaction vessels and stirbars used to obtain optimal and consistent results for the bromochlorination of homoprenol. During the reaction, stirring should not drop below 1500 rpm, and a strong vortex should be maintained.

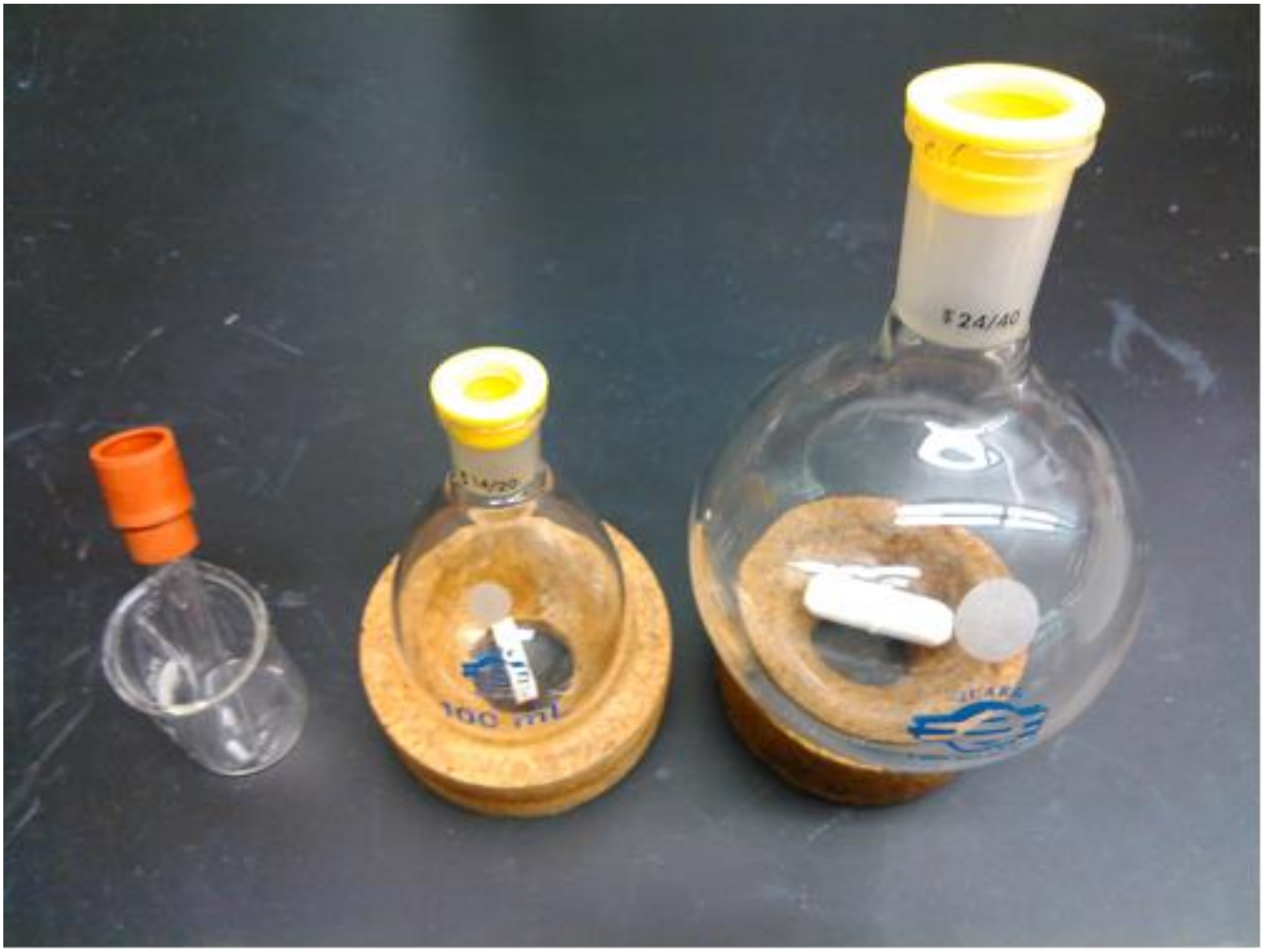

Left: A 10 x 75 mm (~3 mL) test tube containing a magnetic stirring flea (*L* = 7 mm, *D* = 2 mm) sealed with a red septum stopper (14/20) used for up to 0.1 mmol scale reactions.
Middle: A 100 mL 14/20 round-bottomed flask containing a rod-shaped magnetic stir bar (*L* = 25 mm, *D* = 5 mm) sealed with a yellow polyethylene stopper (14/20) used for up to 2 mmol scale reactions.
Right: A 500 mL 24/40 round-bottomed flask containing a rod-shaped magnetic stir bar (*L* = 37 mm, *D* = 10 mm) sealed with a yellow polyethylene stopper (24/40) used for up to 10 mmol scale reactions.

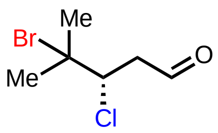

(−)-(S**)-4-Bromo-3-chloro-4-methylpentanal** (**3**): The aldehyde **3** was made from the corresponding alcohol **6** according to the following Dess–Martin oxidation procedure on 3 mmol scale. A 100 mL round-bottomed flask was charged with substrate alcohol **6** (643.7 mg, 2.986 mmol, 1 equiv), $NaHCO_3$ (1.26 g, 5 equiv), and DCM (30 mL, not rigorously dried). The pale yellow homogeneous solution was cooled to 0 °C, and Dess–Martin periodinane (1.53 g, 1.2 equiv) was added all at once. The reaction was allowed to warm slowly to rt. After 90 minutes, TLC analysis indicated full conversion. The reaction was quenched by adding 10 mL saturated aq. $Na_2S_2O_3$ and 10 mL saturated aq. $NaHCO_3$, and was stirred vigorously for 15 minutes. The reaction was partitioned between 30 mL DCM and 30 mL water, and the aqueous layer was extracted with DCM 3 times. The combined organic layers were dried over Na_2SO_4, and the solvent was removed by rotary evaporation at room temperature at a vacuum no stronger than 200 torr. Residual DCM was removed by azeotroping with pentane, again at a vacuum no stronger than 200 torr. This yielded the crude product aldehyde **3** as a yellow oil (605.7 mg, 2.837 mmol, 95% yield). ^{1}H NMR analysis indicated >97% purity by weight with respect to residual solvent. The crude aldehyde **3** was taken onto the next step without further purification.

Physical properties: yellow oil. Somewhat volatile; b.p. not measured.
R_f = 0.27 (silica gel, 10% ethyl acetate / hexanes, anisaldehyde stain);
IR (film) ν_{max} 2978, 2933, 2837, 2729, 1725 (str), 1456, 1388, 1371, 1251, 1102, 1039, 1014, 961, 747, 669, 575 cm^{-1};
^{1}H NMR (600 MHz, $CDCl_3$): 9.80 (d, 1H, *J* = 2.1 Hz), 4.62 (dd, 1H, *J* = 10.3, 2.3 Hz), 3.41 (dd, 1H, *J* = 17.8, 2.3 Hz), 3.03 (ddd, 1H, *J* = 17.8, 10.4, 2.3 Hz), 1.937 (s, 3H), 1.796 (s, 3H);
^{13}C NMR (150 MHz, $CDCl_3$): 197.8, 66.1, 63.0, 49.0, 33.1, 28.0;
GC/MS (CI): calcd. for $C_6H_{10}O^{79}Br^{35}Cl[CH_2CH_3OH][NH_4]^+$: 276.0366, found 276.0359;
$[\alpha]_D^{23}$ = −48.3 (c = 1.0, $CHCl_3$).

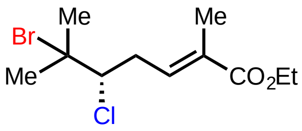

(−)-Ethyl (*S*,*E*)-6-bromo-5-chloro-2,6-dimethylhept-2-enoate (**S12**): The *E*-unsaturated ester **S12** was made from the corresponding aldehyde **3** via the Horner–Wadsworth–Emmons reaction on 2.7 mmol scale according to the following procedure. A dried 50 mL round-bottom flask under argon atmosphere was charged with triethyl 2-phosphonopropionate (**9**, 0.868 mL, 1.5 equiv) and dry THF (10 mL) and cooled to 0 °C. A solution of *n*-BuLi (2.2 M in hexanes, 1.47 mL, 1.2 equiv) was added dropwise over 2 minutes. The ice bath was removed and the solution was allowed to warm to room temperature. After 1 hour, dry acetonitrile (8 mL) was added all at once (polar solvents such as acetonitrile lead to greater *E/Z* selectivity). Then, the substrate aldehyde **3** (575.8 mg, 2.700 mmol, 1 equiv) was dissolved in 2 mL acetonitrile and added dropwise over 30 seconds to the solution of lithiated phosphonate. Exactly 5.0 minutes later, the reaction was quenched by the rapid addition of 10 mL 1 M aq. HCl. TLC analysis at this stage indicated complete conversion of the aldehyde. The reaction was partitioned between 50 mL ether and 50 mL water. The aqueous layer was extracted with ether 3 times; the combined organic layers were washed once with brine, dried over Na_2SO_4, and solvent was removed by rotary evaporation to yield the crude oil. ^{1}H NMR analysis at this stage revealed a 14:1 *E/Z* ratio. The crude oil was purified by column chromatography (silica gel, 5% ethyl acetate/hexanes) to yield the product *E*-unsaturated ester **S12** (529.1 mg, 1.78 mmol, 66% yield). ^{1}H NMR analysis revealed >50:1 *E/Z* ratio for the purified material.

Physical properties: colorless oil.

R_f = 0.38 (silica gel, 5% ethyl acetate / hexanes, anisaldehyde stain); minor *Z* isomer R_f = 0.47 under the same conditions;

IR (film) ν_{max} 2980, 2934, 1709 (str), 1651, 1456, 1388, 1370, 1286, 1251, 1191, 1118, 1103, 1081, 1032, 744, 681, 669, 588 cm^{-1};

^{1}H NMR (600 MHz, $CDCl_3$): 6.83 (ddq, 1H, *J* = 7.9, 6.5, 1.6 Hz), 4.21 (qd, 2H, *J* = 7.2, 0.8 Hz), 4.11 (dd, 1H, *J* = 10.9, 2.1 Hz), 3.22 (ddq, 1H, *J* = 15.7, 6.4, 1.0 Hz), 2.64 (dddd, 1H, *J* = 18.7, 10.9, 7.9, 0.6 Hz), 1.940 (s, 3H), 1.892 (q, 3H, *J* = 0.9 Hz), 1.815 (s, 3H), 1.31 (t, 3H, *J* = 7.1 Hz);

^{13}C NMR (150 MHz, $CDCl_3$): 167.7, 137.2, 130.4, 70.1, 67.3, 60.7, 34.4, 33.6, 27.7, 14.3, 12.8;

GC/MS (CI): calcd. for $C_{11}H_{18}O_2{}^{79}Br^{35}Cl[NH_4]^+$: 314.0522, found 314.0513;

$[\alpha]_D^{23}$ = −34.0 (c = 1.0, $CHCl_3$).

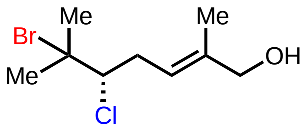

(−)-(*S*,*E*)-6-Bromo-5-chloro-2,6-dimethylhept-2-en-1-ol (**10**): The allylic alcohol **10** was made from the corresponding unsaturated ester **S12** on 1.7 mmol scale according to the following DiBAl−H reduction procedure. A dry 50 mL round-bottomed flask was charged with unsaturated ester **S12** (500.2 mg, 1.681 mmol, 1 equiv) as a solution in dry DCM (8 mL) under argon atmosphere. The solution was cooled to −78 °C. DiBAl−H (0.61 M in hexanes, 8.3 mL, 3 equiv) was added dropwise over 2 minutes. TLC analysis after 20 minutes indicated full conversion. The reaction was quenched by adding 10 mL saturated aq. potassium sodium tartrate (Rochelle's salt) at −78 °C. The suspension was allowed to warm to room temperature with vigorous stirring. After 2 hours, the clear biphasic mixture was extracted 3 times with DCM, the combined organic layers were dried over Na_2SO_4, and solvent was removed by rotary evaporation. The crude oil was purified by column chromatography (silica gel, 25% ethyl acetate/hexanes) to yield the product allylic alcohol **10** (421.3 mg, 1.65 mmol, 98% yield). ^{1}H NMR analysis indicated a 9:1 mixture of constitutionally isomeric bromochlorides favoring that depicted above.

Physical properties: colorless oil.

R_f = 0.42 (silica gel, 25% ethyl acetate / hexanes, anisaldehyde stain);

IR (film) ν_{max} 3320 (br), 2978, 2929, 2863, 1738, 1456, 1387, 1370, 1263, 1217, 1101, 1065, 1007, 964, 930, 857, 812, 780, 669, 583 cm^{-1};

^{1}H NMR (600 MHz, $CDCl_3$): 5.57 (tq, 1H, *J* = 7.0, 1.3 Hz), 4.05 (d, 2H, *J* = 4.6 Hz), 4.01 (dd, 1H, *J* = 10.9, 1.9 Hz), 3.12 (ddq, 1H, *J* = 15.1, 6.7, 0.9 Hz), 2.47 (dddq, 1H, *J* = 18.2, 10.9, 7.4, 0.6 Hz), 1.930 (s, 3H), 1.805 (s, 3H), 1.718 (s, 3H), 1.47 (t, 1H, *J* = 5.6 Hz);

^{13}C NMR (150 MHz, $CDCl_3$): 137.8, 121.6, 71.7, 68.5, 67.9, 33.6, 33.3, 27.8, 14.0;

GC/MS (CI): calcd. for $C_9H_{16}O^{79}Br^{35}Cl[NH_4]^+$: 272.0417, found 272.0412;

$[\alpha]_D^{23}$ = −37.9 (c = 1.0, $CHCl_3$).

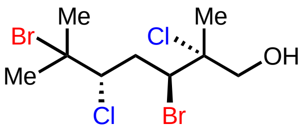

(−)-(2*R*,3*S*,5*S*)-3,6-Dibromo-2,5-dichloro-2,6-dimethylheptan-1-ol (**11**): The (*R,S,S*)-tetrahalide **11** was made from the allylic alcohol **10** on 1.5 mmol scale according to the following bromochlorination procedure. Dry hexanes (40 mL) were added to a dried 100 mL round-bottomed flask containing a 24 mm rod-shaped stirbar under N_2 atmosphere. Solid $ClTi(OiPr)_3$ (0.403 mL, 1.1 equiv) was melted at 60 °C and added as a neat, viscous liquid by syringe using a long, thick (20 gauge) metal needle. $Ti(OiPr)_4$ (89 µL, 0.30 mmol, 0.2 equiv) was added as a neat liquid. Substrate allylic alcohol **10** (393.7 mg, 1.540 mmol) was added as a solution in 10 mL hexanes. With vigorous stirring, (*S,R*)-**4** (66 mg, 0.15 mmol, 0.1 equiv) was added dropwise over 1 minute as a solution in 10 mL hexanes. The pale yellow homogeneous solution was brought to −15 °C in a cryocool. The septum and N_2 balloon were removed, briefly exposing the reaction to ambient air. *N*-Bromosuccinimide (300 mg, 1.7 mmol, 1.2 equiv) was quickly added, and the reaction was capped with a polyethylene yellow stopper. The reaction was stirred at 1500 rpm, maintaining a strong vortex for 42 hours. TLC analysis at this point indicated incomplete conversion. The reaction was quenched with 10 mL 1 M aq. Na_2SO_3 and allowed to warm to room temperature. Then, 10 mL ether and 10 mL conc HCl were added, and the mixture was stirred vigorously for 15 minutes. The now clear layers were separated, the aqueous layer was washed 2 times with ether, and the combined organic layers were washed with 20 mL saturated aqueous $NaHCO_3$. The combined organic layers were dried over Na_2SO_4 and solvent was removed by rotary evaporation yielding the crude tetrahalide **11** as a grainy yellow oil. ^{1}H NMR at this stage indicated no dibromide contamination, so the NaI de-dibromination step was omitted. The recovered starting material **10** and product tetrahalide **11** were both isolated by column chromatography (silica gel, 10% to 25% ethyl acetate/hexanes). The recovered starting material **10** (52.2 mg, 0.20 mmol, 13% yield) was found to have a 1.6:1 constitutional isomeric bromochloride ratio on the left hand side by ^{1}H NMR analysis. The product tetrahalide **11** was isolated as a pale yellow waxy powder (434.2 mg, 1.171 mmol, 76% yield), and was found to have an 18:1 constitutional isomeric bromochloride ratio on the left hand side, and >20:1 diastereoselectivity on the right hand side for the 1,3-*anti* product by ^{1}H NMR analysis.

Physical properties: pale yellow waxy powder, m.p. = 73-78 °C.

R_f = 0.35 (silica gel, 10% ethyl acetate / hexanes, anisaldehyde stain);

IR (film) ν_{max} 3375 (br), 2978, 2933, 2873, 1621, 1455, 1423, 1387, 1372, 1279, 1219, 1103, 1055, 967, 921, 720, 681, 669, 650, 589 cm^{-1};

^{1}H NMR (600 MHz, $CDCl_3$): 4.69 (dd, 1H, *J* = 1.5, 11.2 Hz), 4.36 (dd, 1H, *J* = 10.9, 1.6 Hz), 4.03 (dd, 1H, *J* = 12.3, 8.6 Hz), 3.83 (dd, 1H, *J* = 12.3, 6.0 Hz), 2.77 (ddd, 1H, *J* = 15.0, 11.2, 1.6 Hz), 2.63 (ddd, 1H, *J* = 15.0, 10.8, 1.6 Hz), 2.14 (dd, 1H, *J* = 8.6, 6.0 Hz), 1.955 (s, 3H), 1.826 (s, 3H), 1.646 (s, 3H);

^{13}C NMR (150 MHz, $CDCl_3$): 76.1 (C–Cl), 70.6, 69.1 (C–Cl), 66.6, 56.2, 39.1, 33.5, 28.7, 21.6;

GC/MS (CI): calcd. for $C_9H_{16}{}^{79}Br_2{}^{35}Cl_2O[NH_4]^+$: 385.9289, found 385.9282;

$[\alpha]_D^{23}$ = −49.9 (c = 1.0, $CHCl_3$).

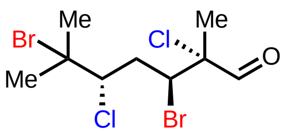

(−)-(2*R*,3*S*,5*S*)-3,6-Dibromo-2,5-dichloro-2,6-dimethylheptanal (**S13**): The aldehyde **S13** was made from the corresponding alcohol **11** according to the following Dess–Martin oxidation procedure on 1 mmol scale. A 100 mL round-bottomed flask was charged with substrate alcohol **11** (395.4 mg, 1.066 mmol, 1 equiv), $NaHCO_3$ (450 mg, 5.3 mmol, 5 equiv) and DCM (10 mL, not rigorously dried). The pale yellow homogeneous solution was cooled to 0 °C, and Dess–Martin periodinane (551 mg, 1.3 mmol, 1.2 equiv) was added all at once. The reaction was allowed to warm slowly to room temperature. After 90 minutes, TLC analysis indicated full conversion. The reaction was quenched by adding 5 mL saturated aq. $Na_2S_2O_3$ and 5 mL saturated aq. $NaHCO_3$, and was stirred vigorously for 15 minutes. The reaction was partitioned between 30 mL DCM and 30 mL water, and the aqueous layer was extracted with DCM 3 times. The combined organic layers were dried over Na_2SO_4, and the solvent was removed by rotary evaporation. The crude pale yellow oil **S13** (392.1 mg, 1.063 mmol, 99% yield) was taken onto the next step without further purification.

Physical properties: pale yellow oil. Apparent instability to silica gel.

R_f = long streak from 0.1 to 0.6 (silica gel, 10% ethyl acetate / hexanes, anisaldehyde stain);

IR (film) ν_{max} 2978, 2933, 2827, 1741 (str), 1455, 1422, 1389, 1372, 1284, 1221, 1103, 1060, 967, 917, 679, 669, 656, 588 cm^{-1};

^{1}H NMR (600 MHz, $CDCl_3$): 9.27 (s, 1H), 4.63 (dd, 1H, *J* = 11.2, 1.7 Hz), 4.34 (dd, 1H, *J* = 10.9, 1.6 Hz), 2.77 (ddd, 1H, *J* = 15.0, 11.3, 1.7 Hz), 2.55 (ddd, 1H, *J* = 15.0, 10.8, 1.7 Hz), 1.962 (s, 3H), 1.831 (s, 3H), 1.776 (s, 3H);

^{13}C NMR (150 MHz, $CDCl_3$): 189.9, 72.1, 68.4, 66.1, 53.4, 37.6, 33.5, 28.6, 18.6;

GC/MS (CI): calcd. for $C_9H_{14}O^{79}Br_2{}^{35}Cl_2[NH_4]^+$: 383.9132, found 383.9127;

$[\alpha]_D^{23}$ = −11.1 (c = 1.0, $CHCl_3$).

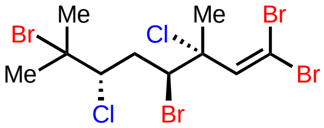

(−)-(3*R*,4*S*,6*S*)-1,1,4,7-Tetrabromo-3,6-dichloro-3,7-dimethyloct-1-ene (**12**): The geminal 1,1-dibromoolefin **12** was made from the corresponding aldehyde **S13** on 1 mmol scale according to the following Ramirez dibromomethylenation procedure. A 50 mL round-bottomed flask was charged with CBr_4 (663 mg, 2 mmol, 2 equiv), PPh_3 (1.05 g, 4 mmol, 4 equiv), and DCM (3 mL, not rigorously dried). The homogeneous mixture was cooled to 0 °C. The substrate aldehyde **S13** (362.5 mg, 0.9827 mmol, 1 equiv) was added as a solution in 2 mL DCM. After 10 minutes, TLC analysis indicated full conversion. While at 0 °C, hexanes (20 mL) were added dropwise to the reaction with vigorous stirring, precipitating out PPh_3O as a viscous gum. The mixture was filtered, collecting the filtrate. The gum was re-dissolved in DCM (5 mL) followed by dropwise hexanes (20 mL) and filtered iteratively 2 more times. The combined filtrate was then filtered again one last time, at which point TLC analysis indicated that the filtrate contained minimal phosphine or phosphine oxide byproducts, and the gum contained no product. The filtrate was collected, and the solvent was removed by rotary evaporation. The obtained crude grainy solid was taken up in hexanes and sonicated to form a fine suspension in hexanes, then purified by column chromatography (silica gel, 100% hexanes). Mixed fractions containing phosphine byproducts were re-subjected to a second column. The product **12** obtained was a viscous colorless oil (429.0 mg, 0.8176 mmol, 83% yield), which contained no phosphine or phosphine oxide byproducts by ^{1}H NMR analysis.

Physical properties: viscous colorless oil. Does not solidify at RT.
R_f = 0.56 (silica gel, 100% hexanes, anisaldehyde stain);
IR (film) ν_{max} 2980, 2933, 1608, 1583, 1455, 1423, 1380, 1278, 1221, 1154, 1103, 1052, 1031, 971, 922, 880, 814, 781, 683, 669, 654, 583 cm^{-1};
^{1}H NMR (600 MHz, $CDCl_3$): 6.94 (s, 1H), 4.90 (dd, 1H, *J* = 11.0, 1.5 Hz), 4.34 (dd, 1H, *J* = 10.9, 1.5 Hz), 2.79 (ddd, 1H, *J* = 15.0, 11.0, 1.6 Hz), 2.56 (ddd, 1H, *J* = 15.0, 10.9, 1.5 Hz), 1.985 (s, 3H), 1.953 (s, 3H), 1.826 (s, 3H);
^{13}C NMR (150 MHz, $CDCl_3$): 139.9, 93.3, 70.9, 69.1, 66.3, 58.9, 39.1, 33.5, 28.7, 26.3;
GC/MS (CI): Parent ion or adduct not seen;
calcd. for $[M – BrCl]^+$: 405.8, found 405.8 with appropriate signature;
calcd. for $[M – HBr]^+$, $C_{10}H_{13}{}^{79}Br_3{}^{35}Cl_2{}^+$: 439.7944, found 439.7934;
$[\alpha]_D^{23}$ = −34.4 (c = 1.0, $CHCl_3$).

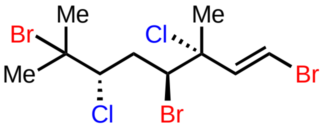

(−)-(3*R*,4*S*,6*S*,*E***)-1,4,7-Tribromo-3,6-dichloro-3,7-dimethyloct-1-ene** (anverene, **1**): The *E*-vinyl bromide of anverene **1** was made from the corresponding 1,1-dibromoolefin **12** on 0.7 mmol scale according to the following Hirao reduction procedure. A 20 mL scintillation vial under ambient air at room temperature was charged with 1,1-dibromoolefin substrate **12** (375.8 mg, 0.716 mmol, 1 equiv), followed by DMF (3 mL, not rigorously dried), Et_3N (0.5 mL, 3.6 mmol, 5 equiv), and finally dimethyl phosphonate (0.27 mL, 2.9 mmol, 4 equiv). The pale yellow cloudy reaction was stirred at room temperature. After 5 minutes, TLC analysis of an aliquot (partitioned between diethyl ether/H_2O) indicated full conversion. The reaction was quenched by partitioning between 50 mL diethyl ether and 50 mL water. The aqueous layer was extracted 3 times with ether. The combined organic layers were washed with 30 mL 1 M aq. LiCl, dried over Na_2SO_4, and the solvent was removed by rotary evaporation. The crude oil, with some water contamination, was taken up in hexanes, dried again over Na_2SO_4, and the solvent was again removed by rotary evaporation. The pale yellow crude oil was purified by column chromatography (silica gel, 100% hexanes) to provide the product **1** as a colorless viscous oil (281.9 mg, 0.632 mmol, 88% yield). ^{1}H NMR analysis indicated a 7:1 mixture of vinyl bromide *E/Z* isomers. The *E/Z* mixture was purified by recrystallization by mixing an equal volume of hexanes (300 μL) into the neat product at room temperature and letting it sit overnight. After two recrystallization batches, 104.2 mg of anverene (**1**) was obtained as a crystalline solid. ^{1}H NMR analysis indicated a >50:1 *E*/*Z* ratio.

Physical properties: white crystalline solid; m.p. = 65-69 °C.

$\mathbf{R_f}$ = 0.50 (silica gel, 100% hexanes, anisaldehyde stain).

IR (film) ν_{max} 3085, 2978, 2932, 1621, 1455, 1423, 1380, 1283, 1221, 1103, 1050, 971, 934, 754, 682, 669, 646, 588 cm^{-1};

^{1}H NMR (600 MHz, $CDCl_3$): 6.59 (d, 1H, *J* = 13.5 Hz), 6.42 (d, 1H, *J* = 13.5 Hz), 4.40 (dd, 1H, *J* = 11.0, 1.6 Hz), 4.34 (dd, 1H, *J* = 1.5, 10.8 Hz), 2.74 (ddd, 1H, *J* = 14.9, 11.0, 1.6 Hz), 2.56 (ddd, 1H, *J* = 14.9, 10.8, 1.6 Hz), 1.944 (s, 3H), 1.82 (overlapping s, 6H);

^{13}C NMR (150 MHz, $CDCl_3$): 139.9, 109.8, 72.0 (C–Cl), 69.3 (C–Cl), 66.6, 59.9, 39.3, 33.5, 28.8, 25.6;

GC/MS (CI): Parent ion or adduct not seen;

calcd. for $[M - BrCl]^+$: 327.9; found 327.9 with appropriate signature;

calcd. for $[M – HBr]^+$, $C_{10}H_{14}{}^{79}Br_2{}^{35}Cl_2{}^+$: 361.8839, found 361.8836;

Initial characterization (Ankisetty et al. 2004)[4]*:* Parent ion not seen; only $[M – HCl]^+$ and $[M – 2HCl]^+$ observed;

$\mathbf{[\alpha]_D^{23}}$ = −35.3 (c = 1.0, $CHCl_3$);

$\mathbf{[\alpha]_D^{23}}$ = −33.9 (c = 0.25, $CHCl_3$);

Initial characterization (Ankisetty et al. 2004)[4]*:* $\mathbf{[\alpha]_D^{25}}$ = −12 (c = 0.25, $CHCl_3$).

4. ^{1}H and ^{13}C NMR spectra

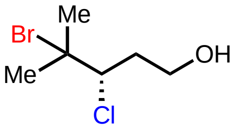

6

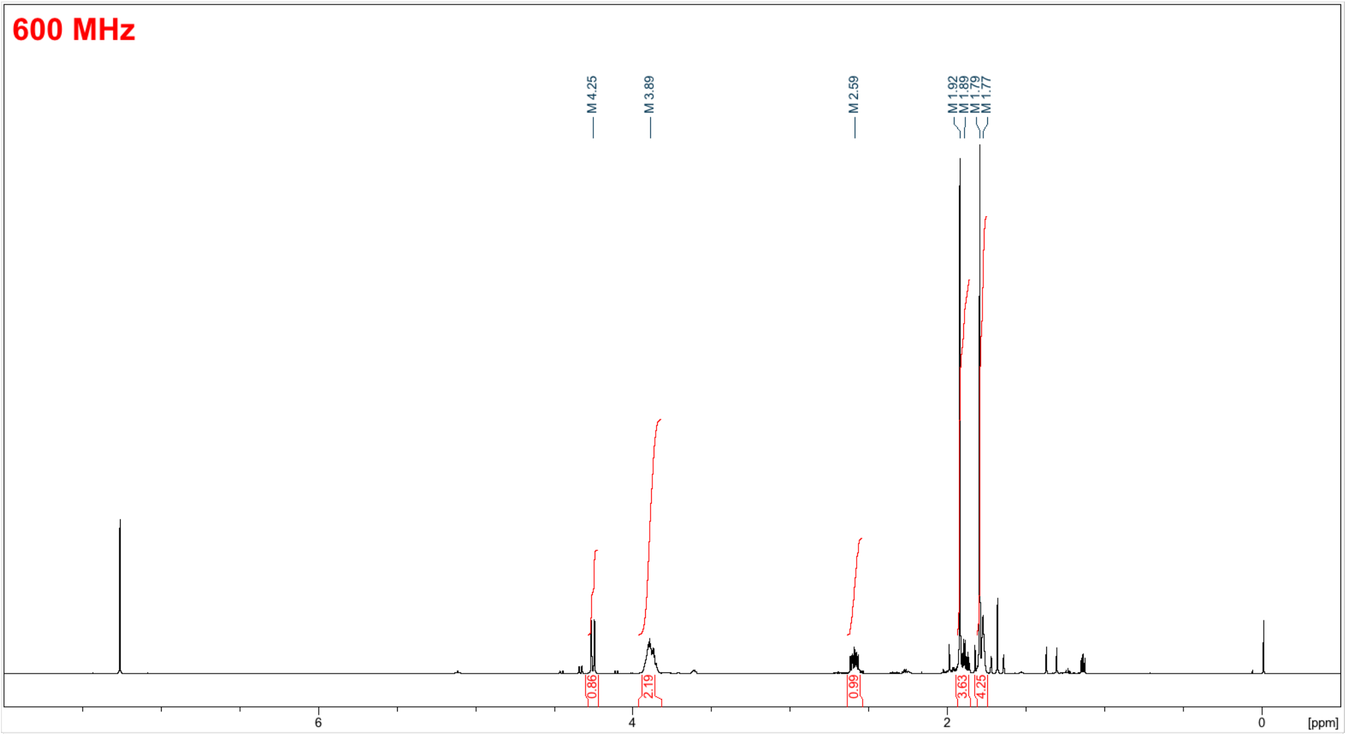

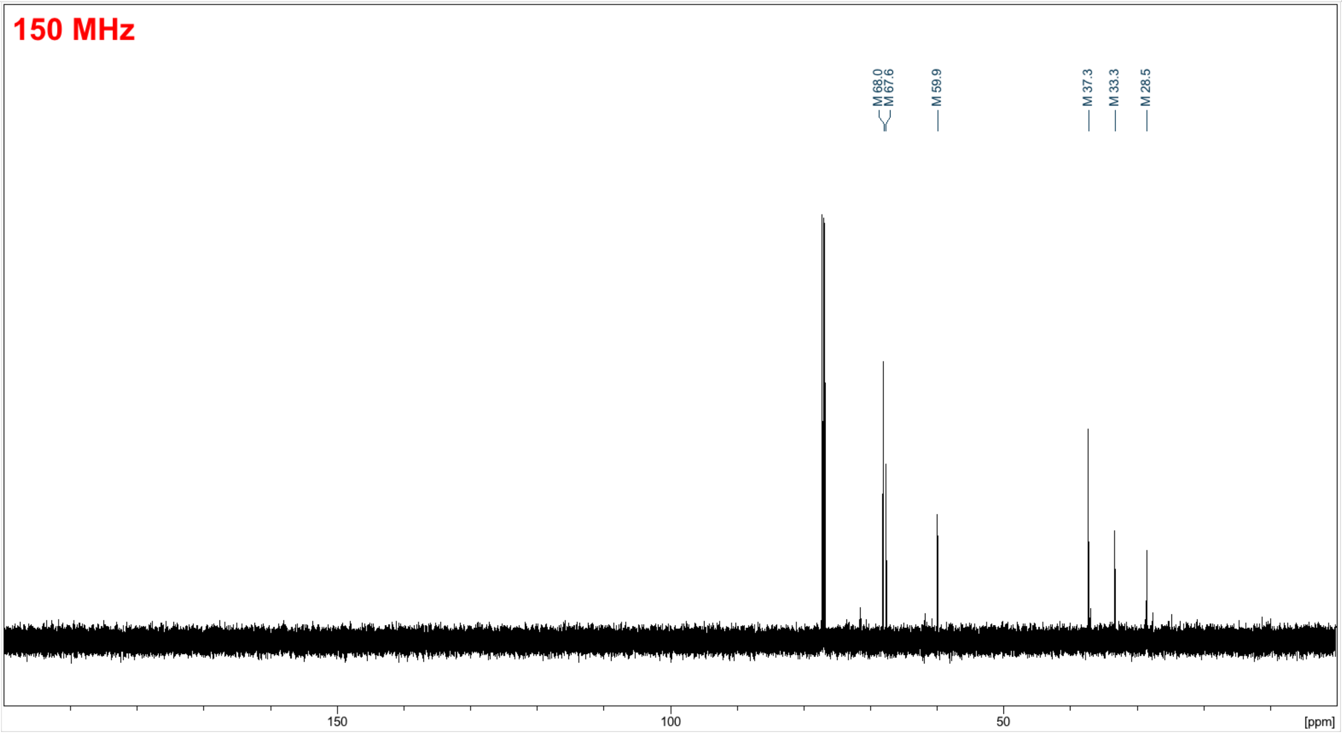

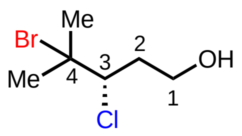

6

HSQC data indicate that C3 (68.0 ppm) is a methine and that C4 (67.6 ppm) is a tertiary halide.

HSQC

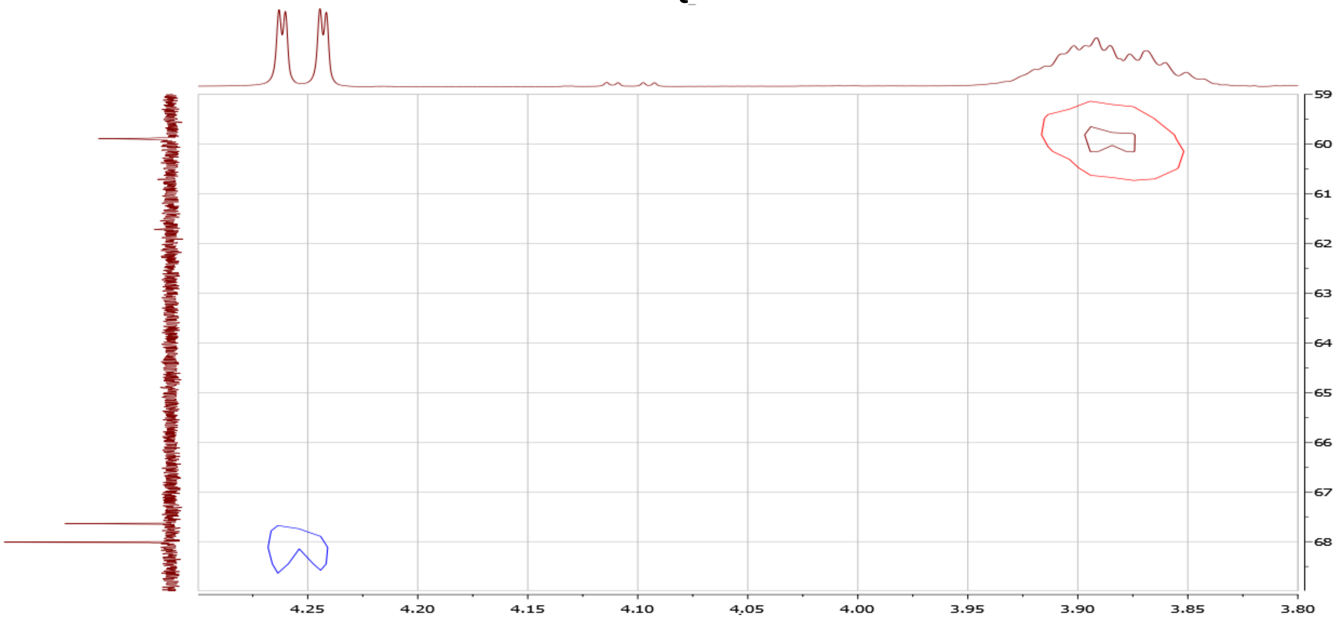

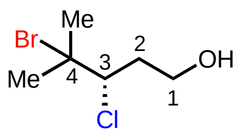

6

Distinct chlorine isotopic shift (Δδ = 10 ppb) observed at C3 [5].

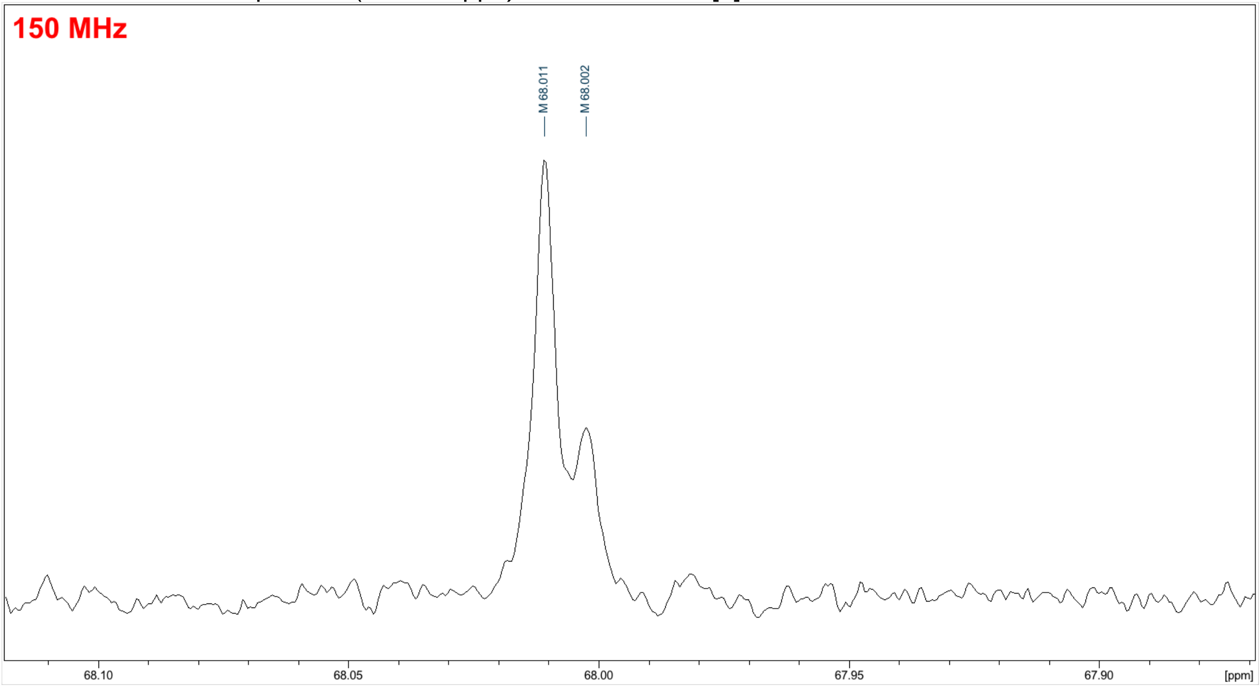

No chlorine isotopic shift observed at C4:

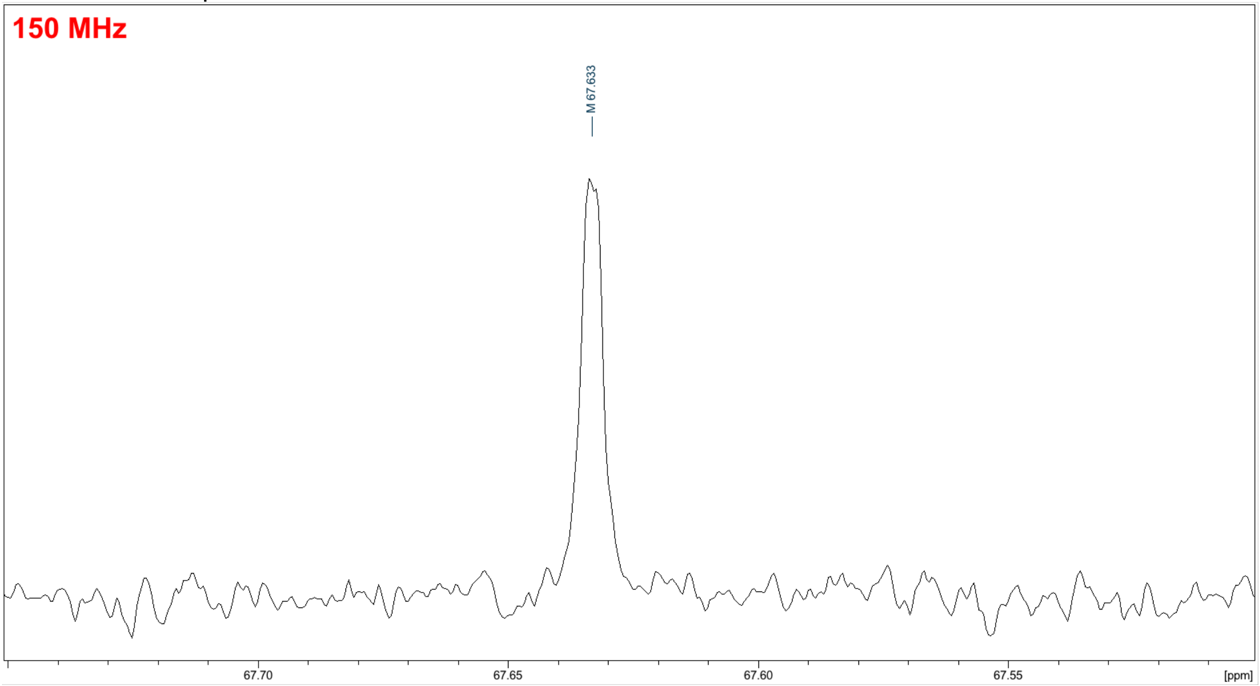

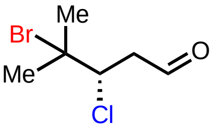

Me
Br
Me
O
Cl

3

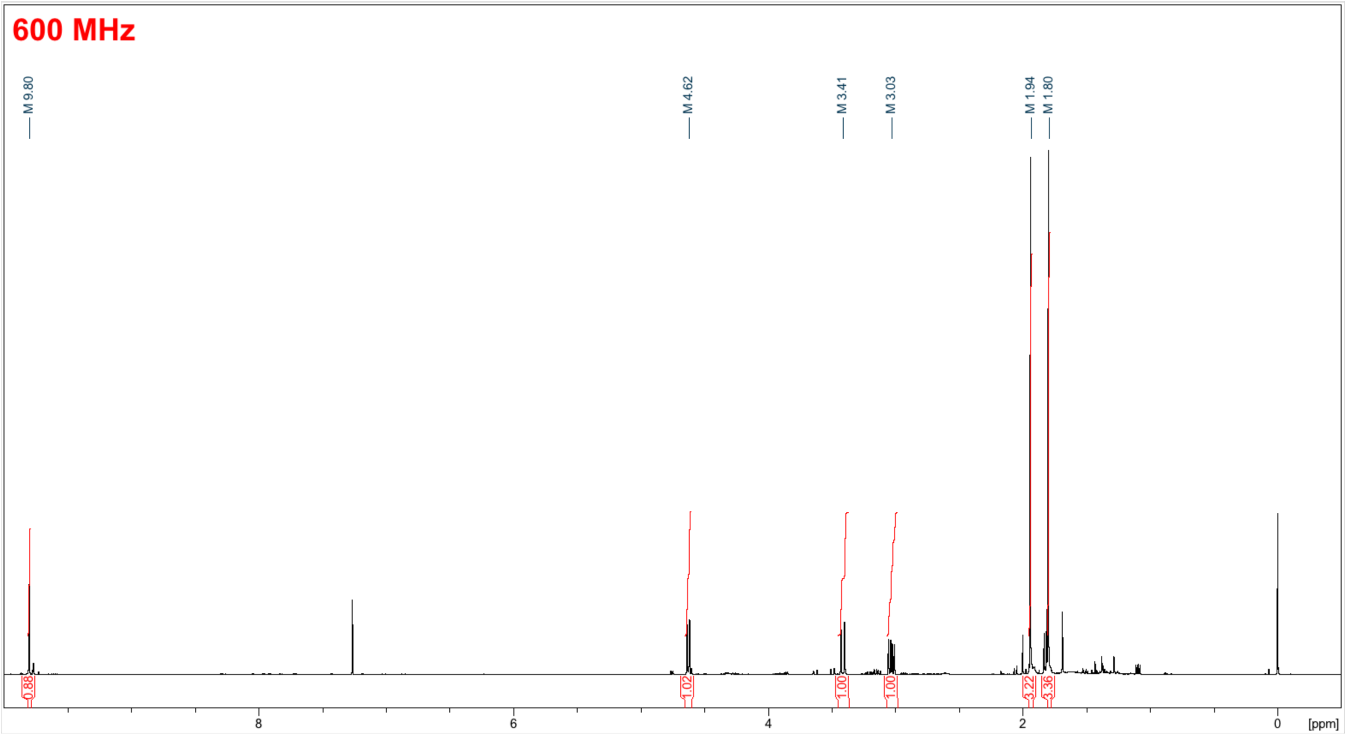

600 MHz
M 9.80
M 4.62
M 3.41
M 3.03
M 1.94
M 1.80
0.88
1.02
1.00
1.00
3.22
3.36
8
6
4
2
0
[ppm]

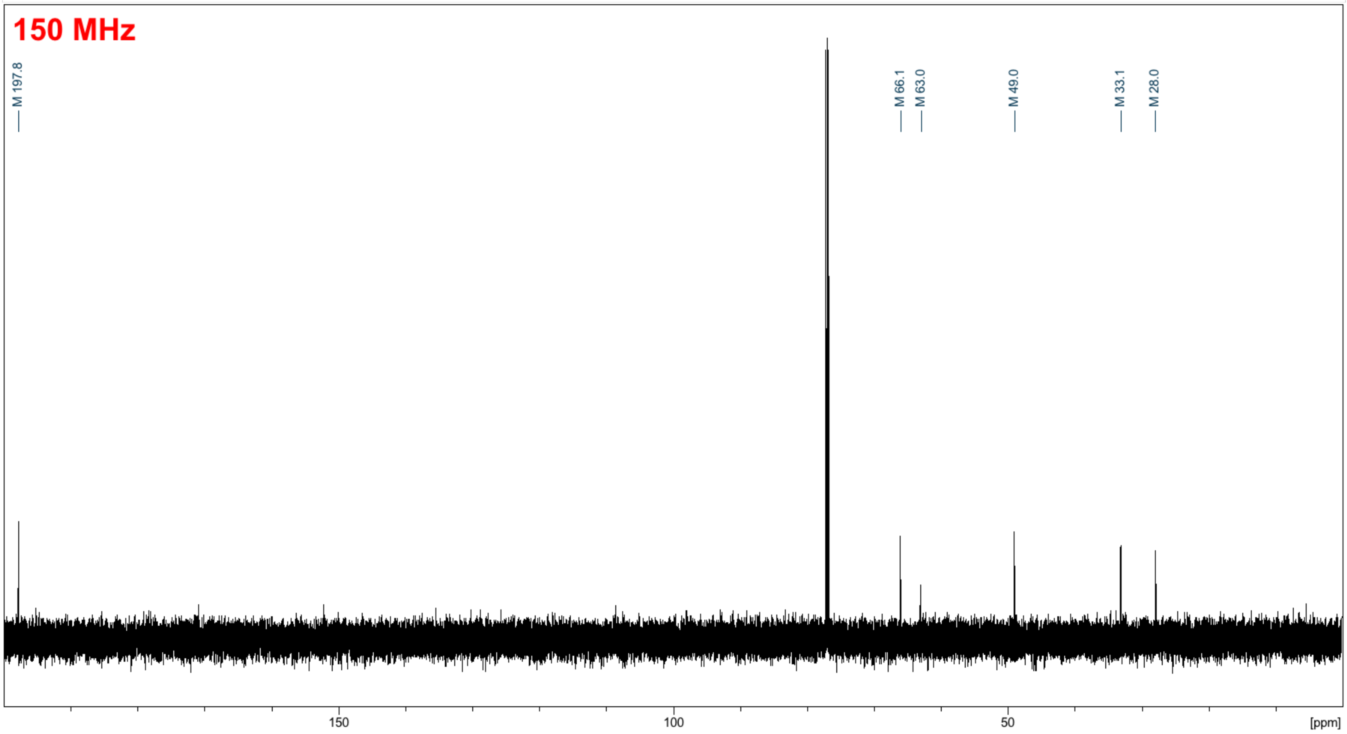

150 MHz
M 197.8
M 66.1
M 63.0
M 49.0
M 33.1
M 28.0
150
100
50
[ppm]

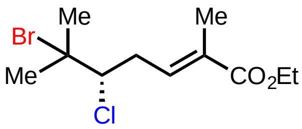
Me
Me
Br
Me
CO2Et
Cl

S12

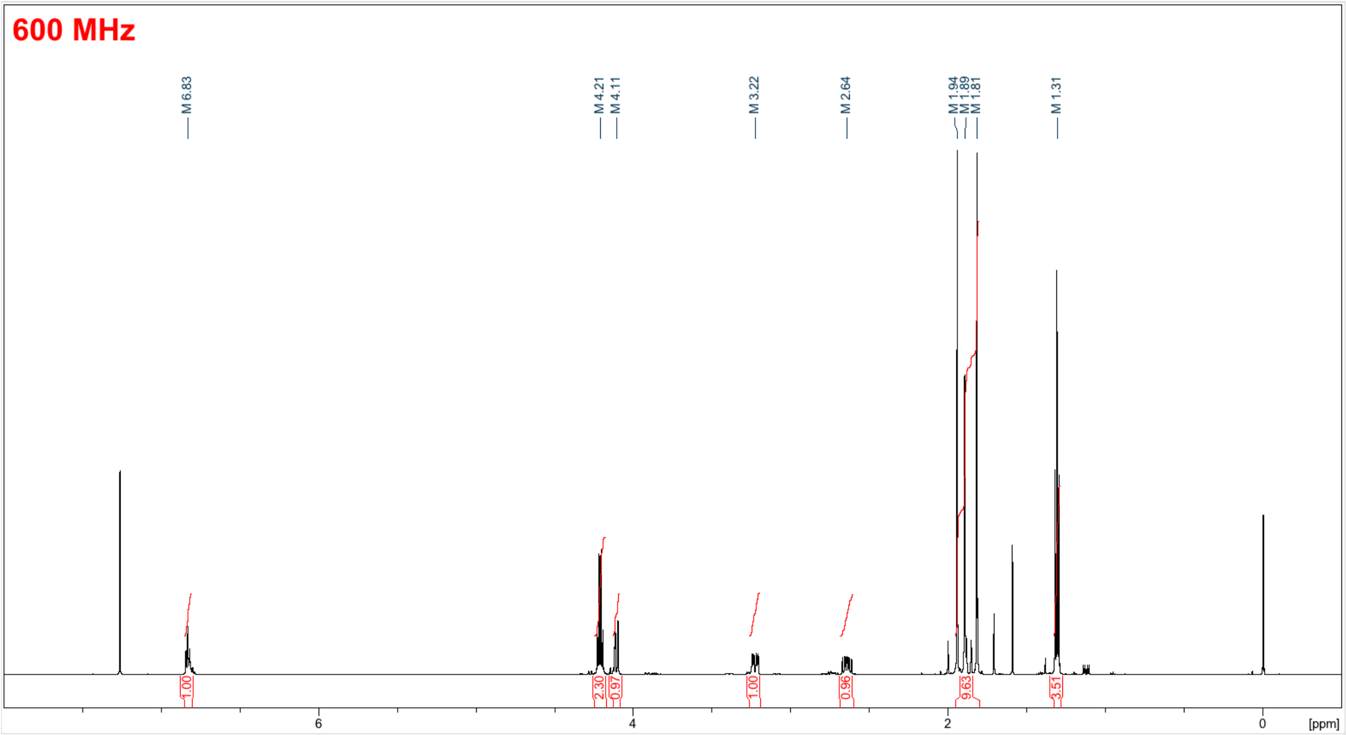
600 MHz
M 6.83
M 4.21
M 4.11
M 3.22
M 2.64
M 1.94
M 1.89
M 1.81
M 1.31
1.00
2.30
0.97
1.00
0.96
9.63
3.51
6
4
2
0
[ppm]

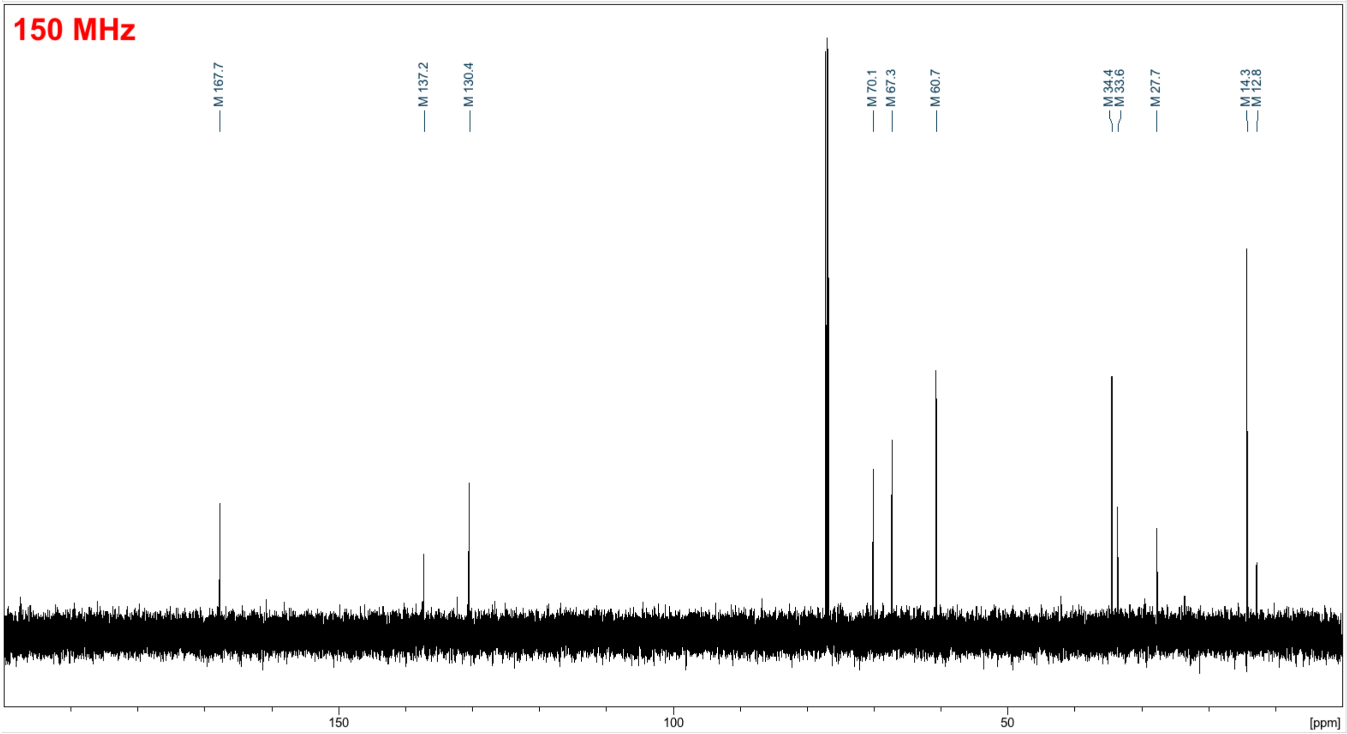
150 MHz
M 167.7
M 137.2
M 130.4
M 70.1
M 67.3
M 60.7
M 34.4
M 33.6
M 27.7
M 14.3
M 12.8
150
100
50
[ppm]

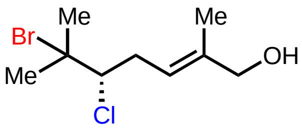

Me
Br
Me
Me
OH
Cl

10

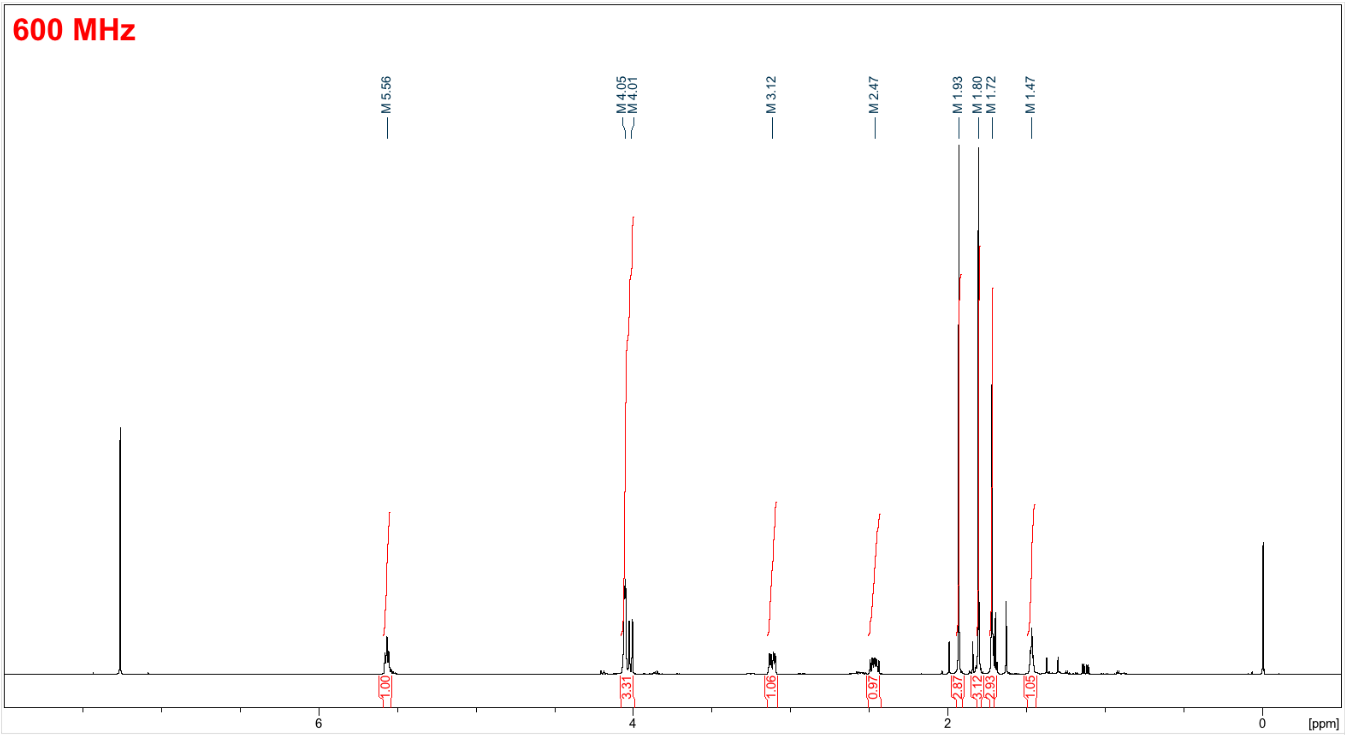

600 MHz
M 5.56
M 4.05
M 4.01
M 3.12
M 2.47
M 1.93
M 1.80
M 1.72
M 1.47
1.00
3.31
1.06
0.97
2.87
3.12
2.93
1.05
6
4
2
0
[ppm]

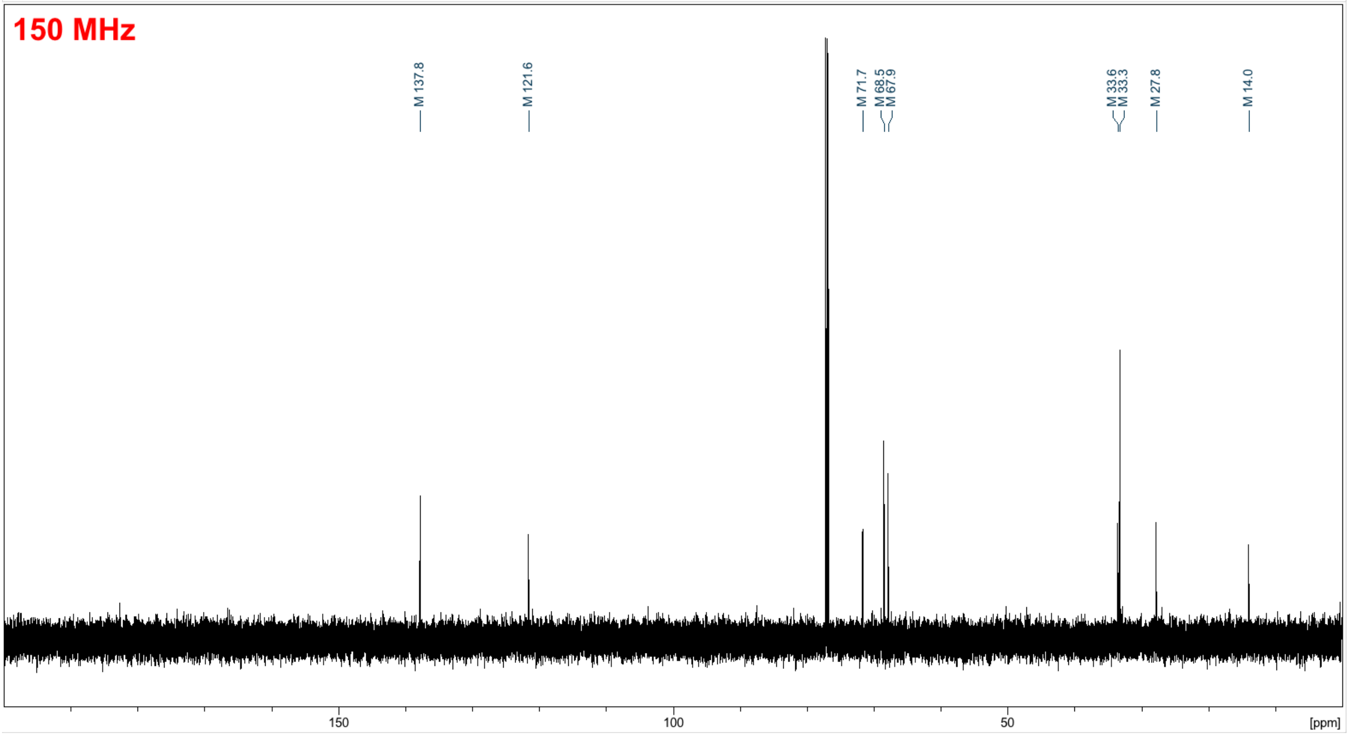

150 MHz
M 137.8
M 121.6
M 71.7
M 68.5
M 67.9
M 33.6
M 33.3
M 27.8
M 14.0
150
100
50
[ppm]

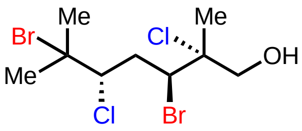
Me
Br
Me
Cl
Me
OH
Cl
Br

11

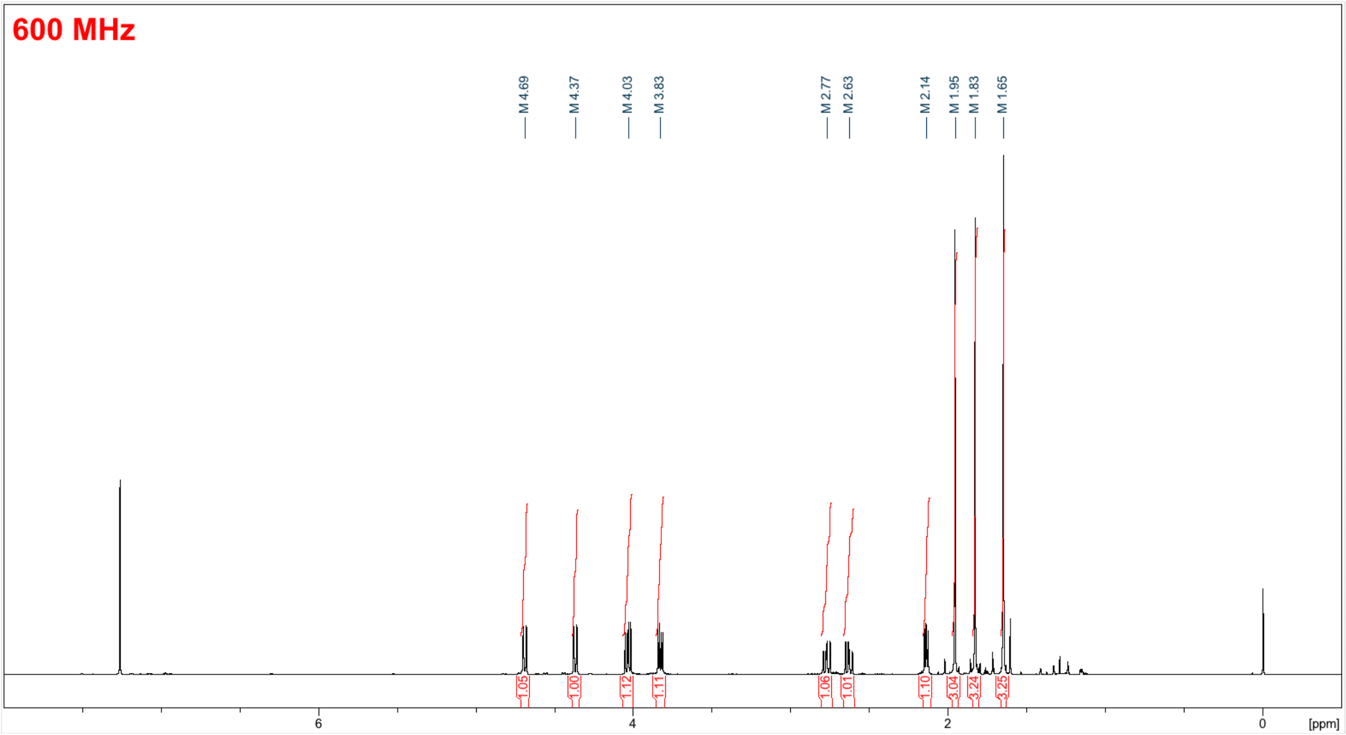
600 MHz
M 4.69
M 4.37
M 4.03
M 3.83
M 2.77
M 2.63
M 2.14
M 1.95
M 1.83
M 1.65
1.05
1.00
1.12
1.11
1.06
1.01
1.10
3.04
3.24
3.25
6
4
2
0
[ppm]

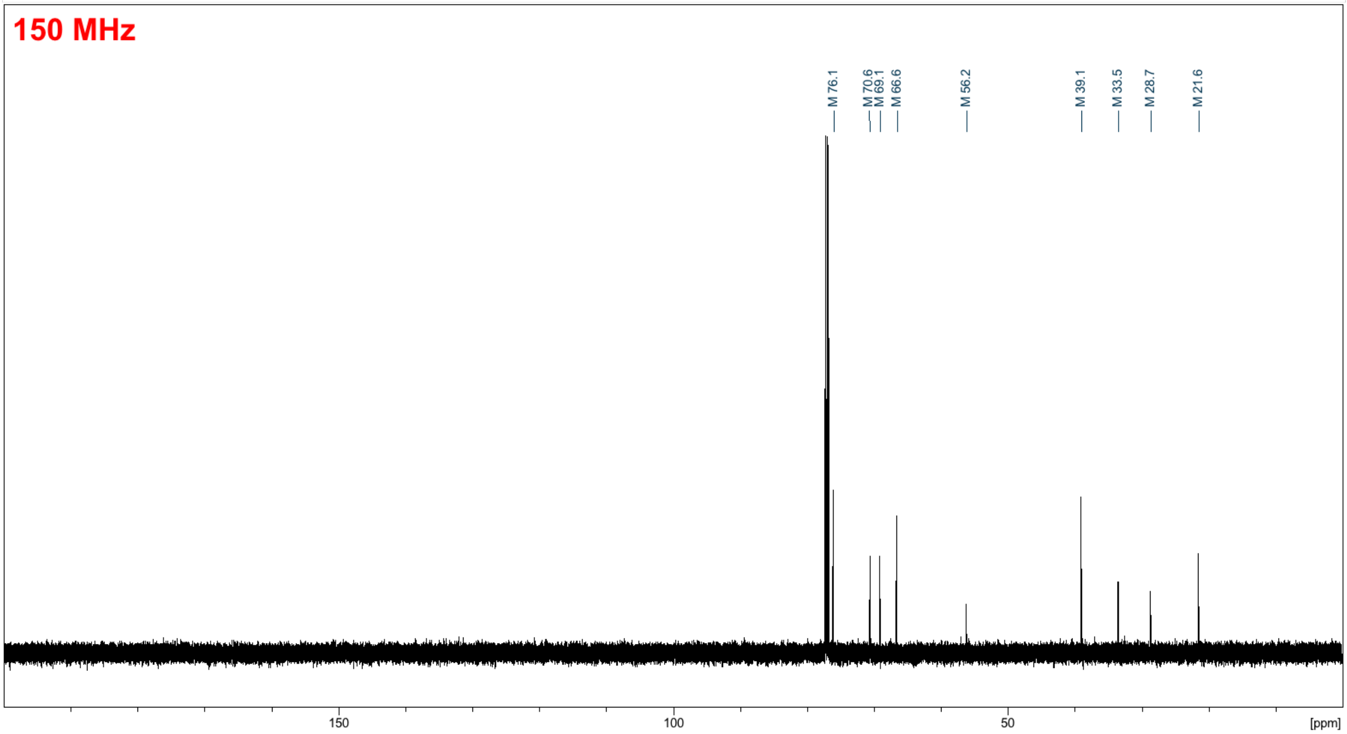
150 MHz
M 76.1
M 70.6
M 69.1
M 66.6
M 56.2
M 39.1
M 33.5
M 28.7
M 21.6
150
100
50
[ppm]

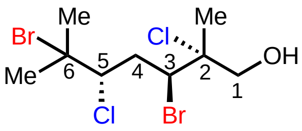

11

HSQC data indicate that C5 (69.1 ppm) and C3 (56.2 ppm) are methines, and that C2 (76.1 ppm) and C6 (66.6 ppm) are tertiary halides.

HSQC

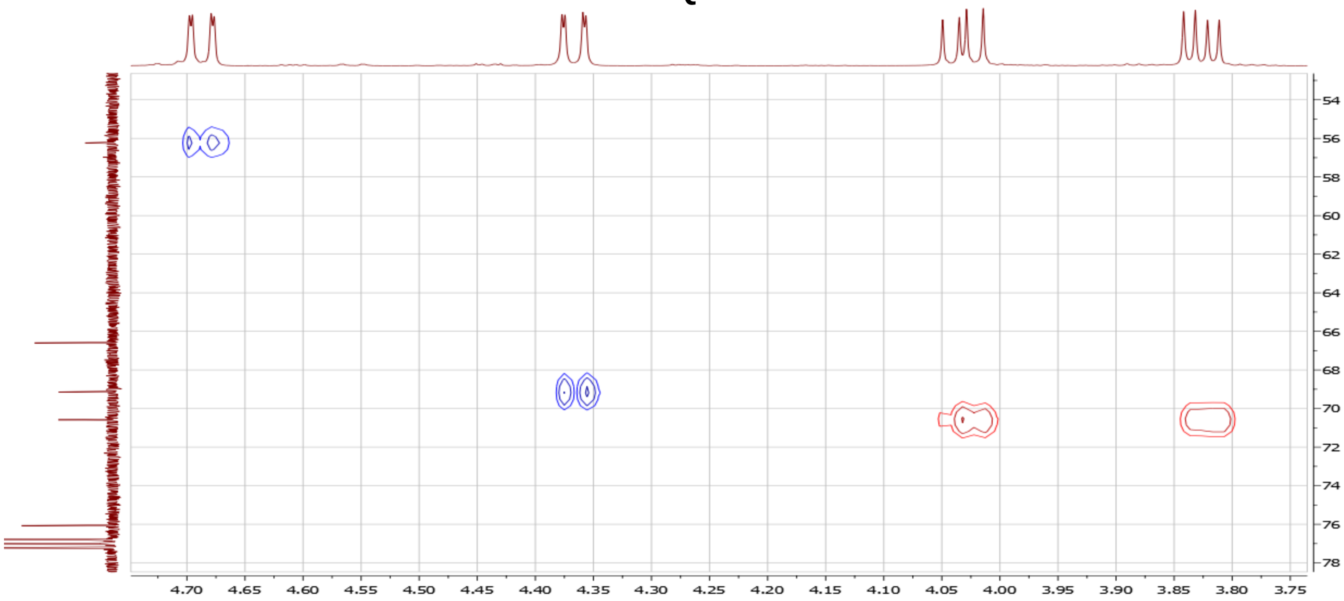

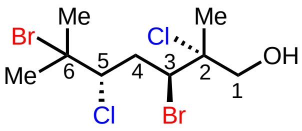

11

Distinct chlorine isotopic shift (Δδ = 11 ppb) observed at C2:

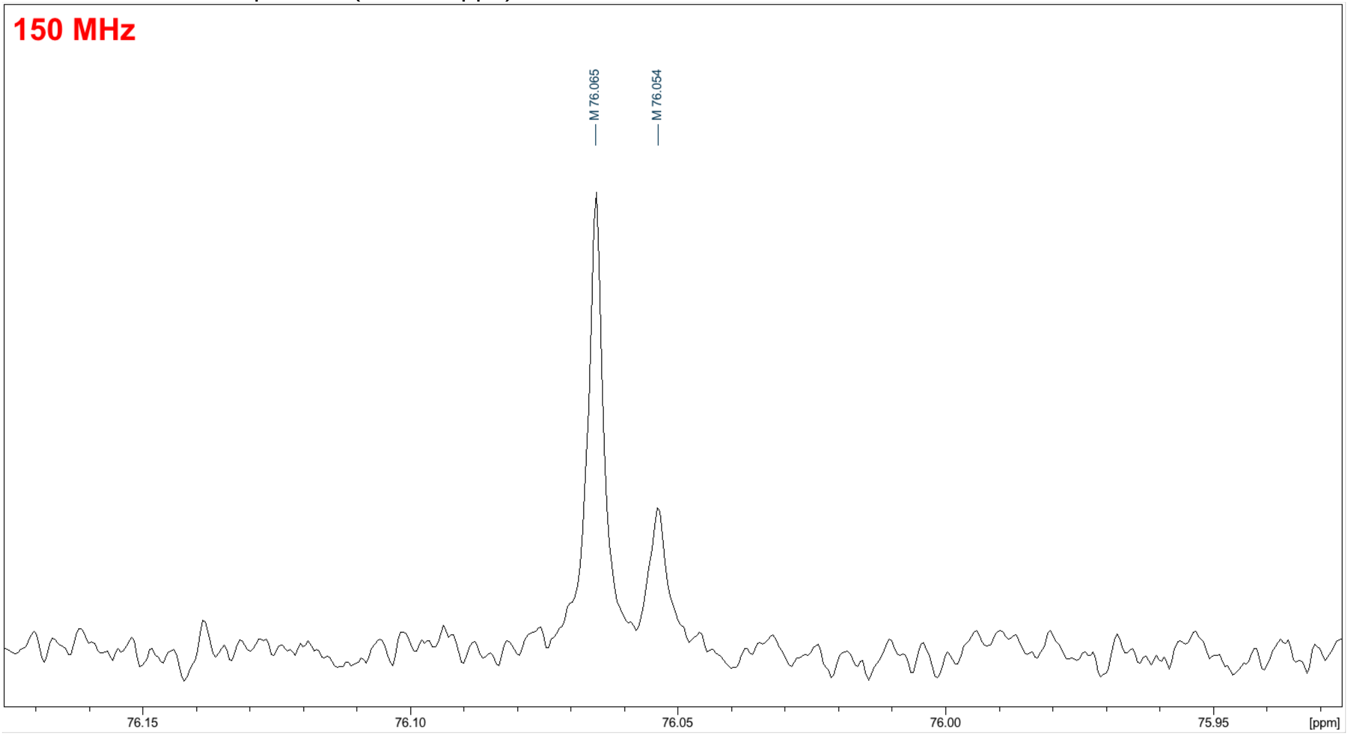

In this compound, the chlorine isotopic shift at C5 is not visible due to broadening:

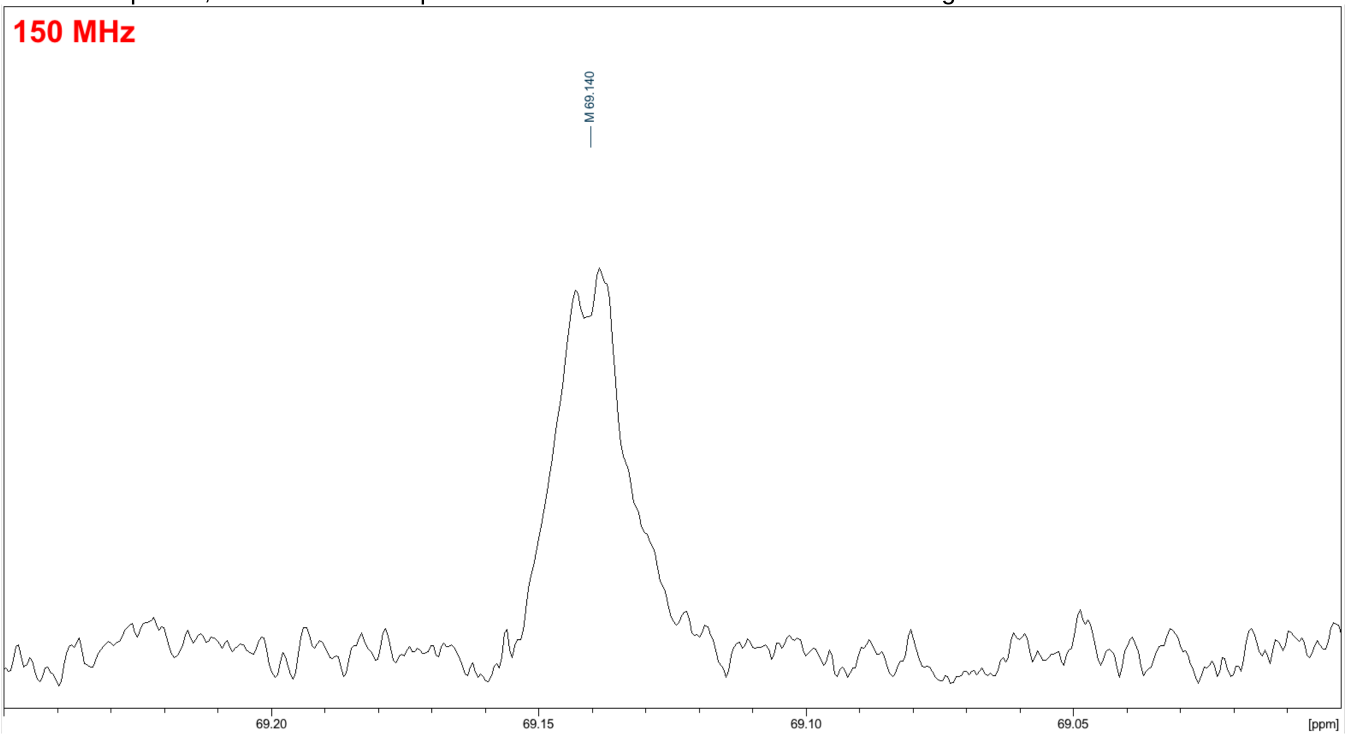

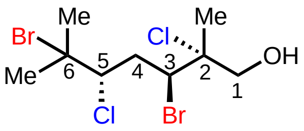

11

No chlorine isotopic shift seen at C3 (note significant broadening at this carbon as well):

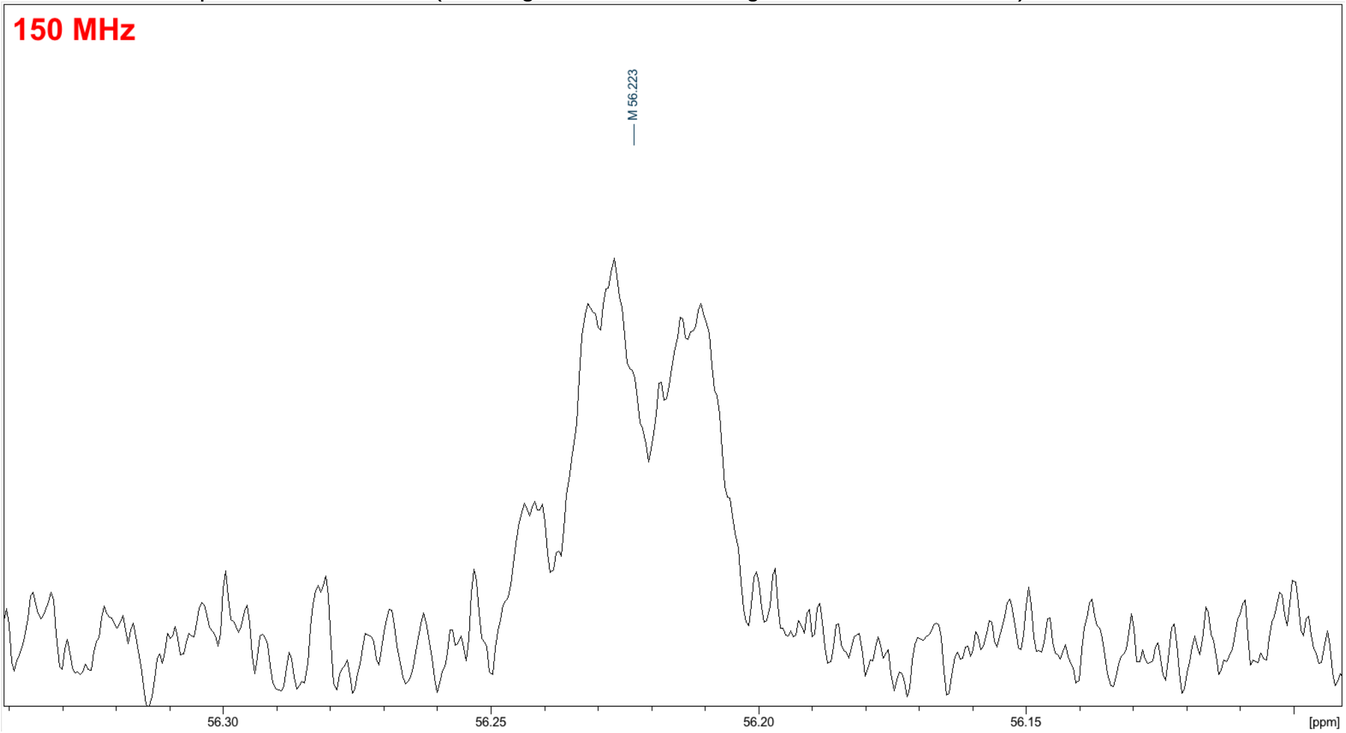

No chlorine isotopic shift seen at C6:

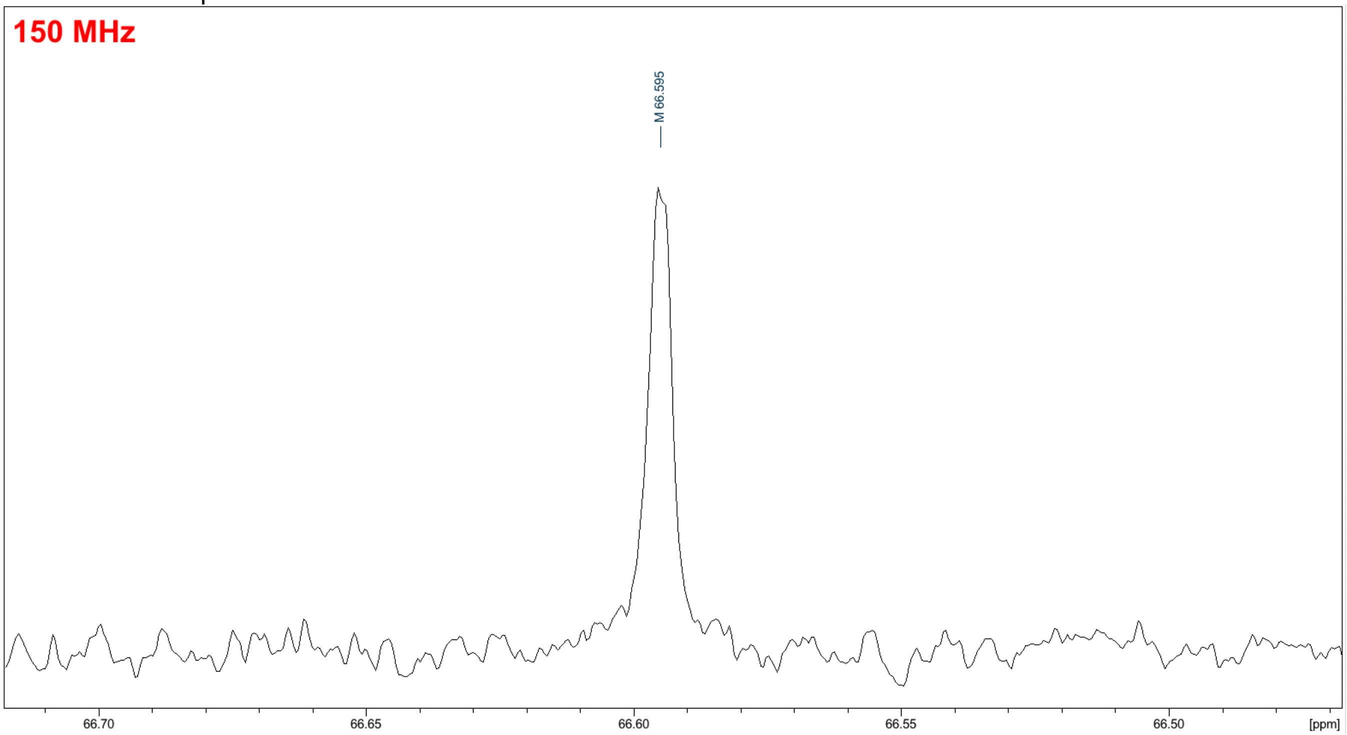

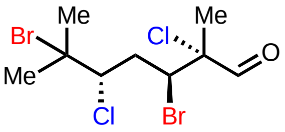
Me
Br
Me
Cl
Me
Cl
O
Cl
Br

S13

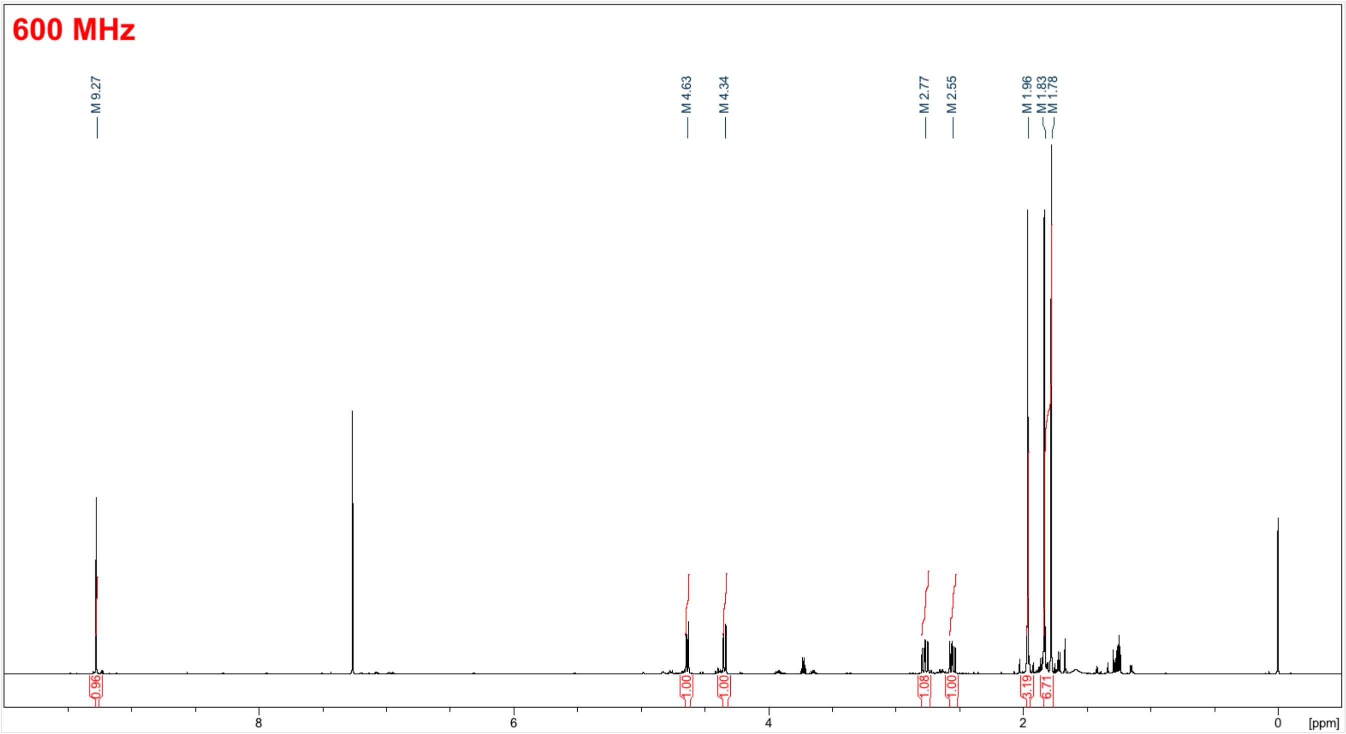
600 MHz
M 9.27
M 4.63
M 4.34
M 2.77
M 2.55
M 1.96
M 1.83
M 1.78
0.96
1.00
1.00
1.08
1.00
3.19
6.71
8
6
4
2
0
[ppm]

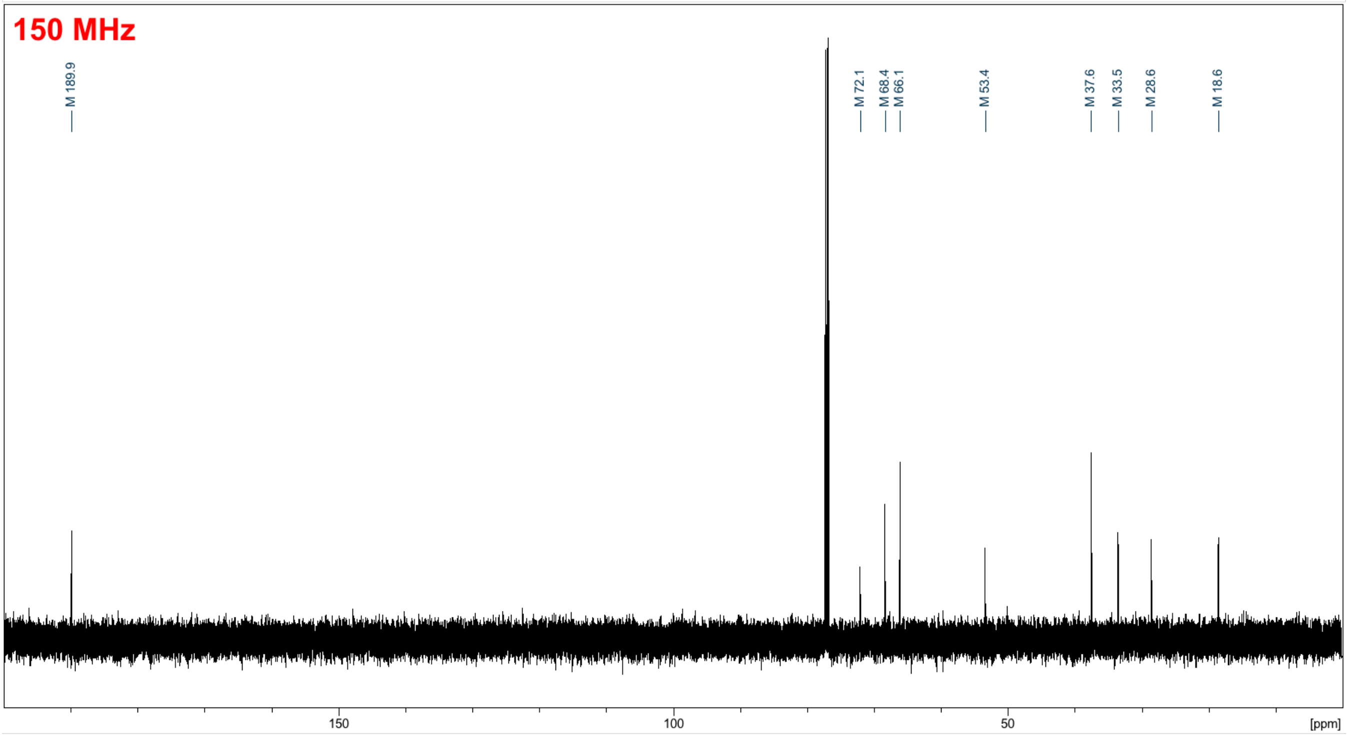
150 MHz
M 189.9
M 72.1
M 68.4
M 66.1
M 53.4
M 37.6
M 33.5
M 28.6
M 18.6
150
100
50
[ppm]

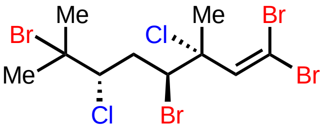
Me
Br
Me
Cl
Cl
Me
Br
Br
Br

12

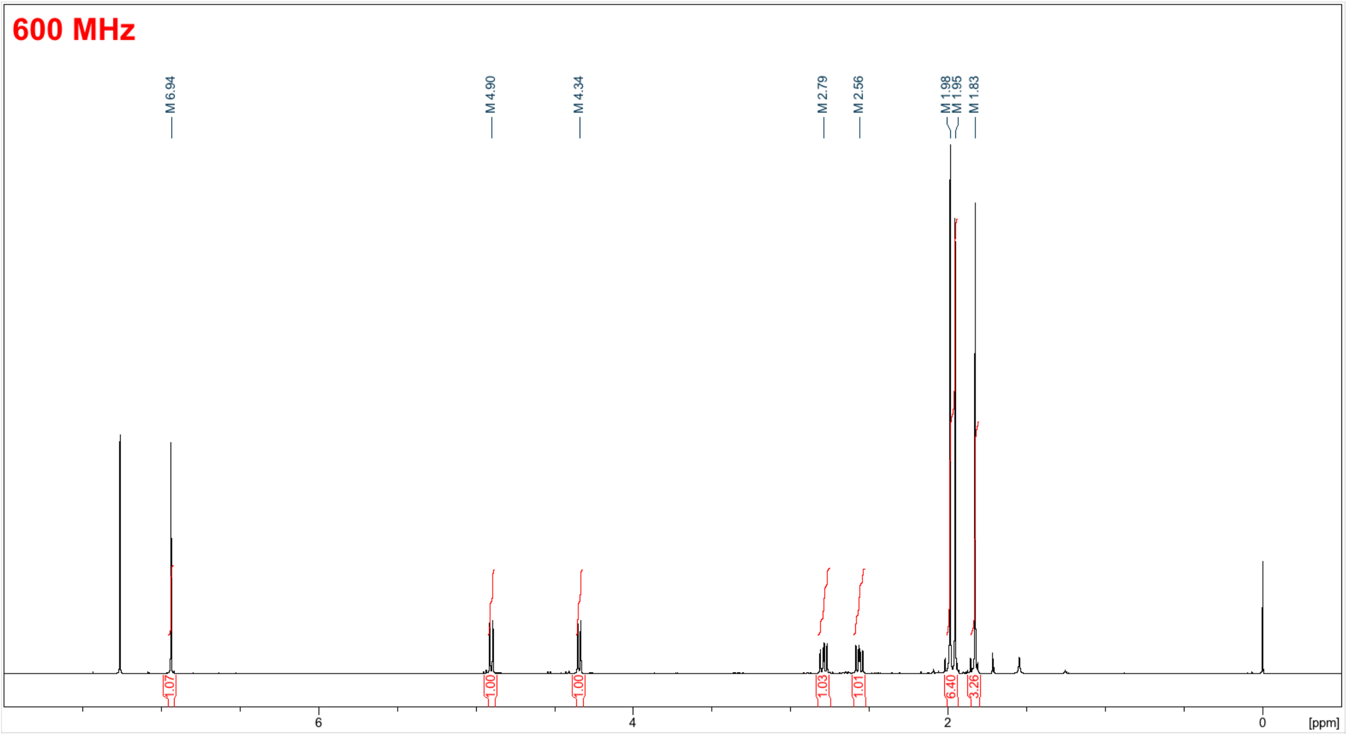
600 MHz
M 6.94
M 4.90
M 4.34
M 2.79
M 2.56
M 1.98
M 1.95
M 1.83
1.07
1.00
1.00
1.03
1.01
6.40
3.26
6
4
2
0
[ppm]

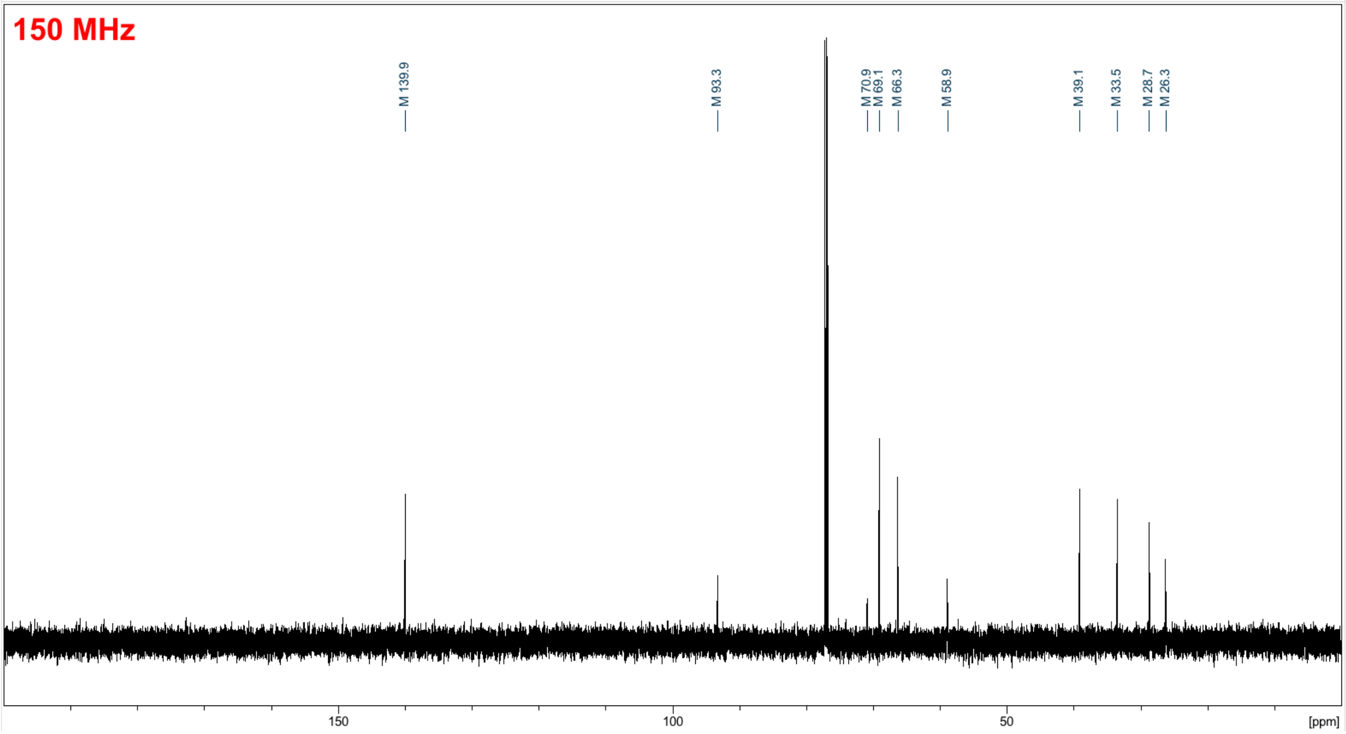
150 MHz
M 139.9
M 93.3
M 70.9
M 69.1
M 66.3
M 58.9
M 39.1
M 33.5
M 28.7
M 26.3
150
100
50
[ppm]

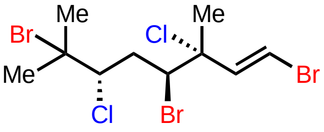
Me
Br
Me
Cl
Cl
Me
Br
Br

1

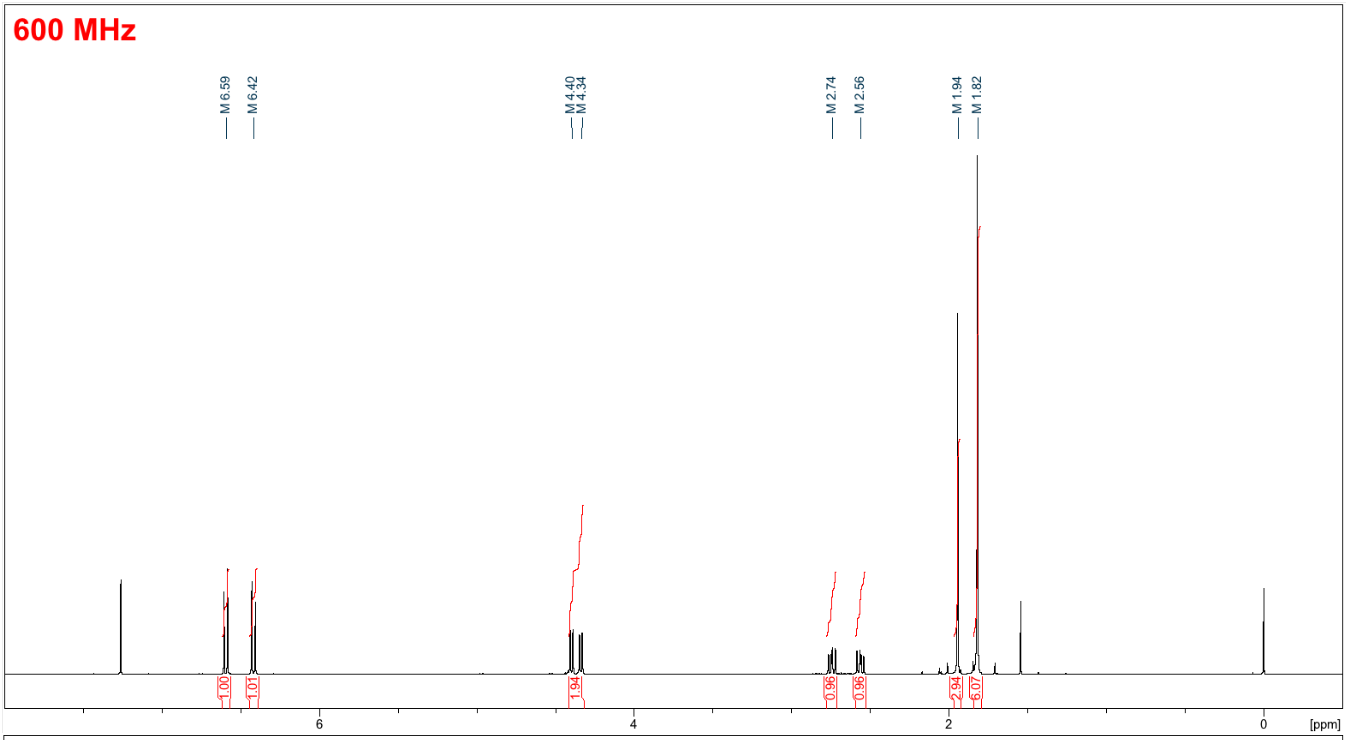
600 MHz
M 6.59
M 6.42
M 4.40
M 4.34
M 2.74
M 2.56
M 1.94
M 1.82
1.00
1.01
1.94
0.96
0.96
2.94
6.07
6
4
2
0
[ppm]

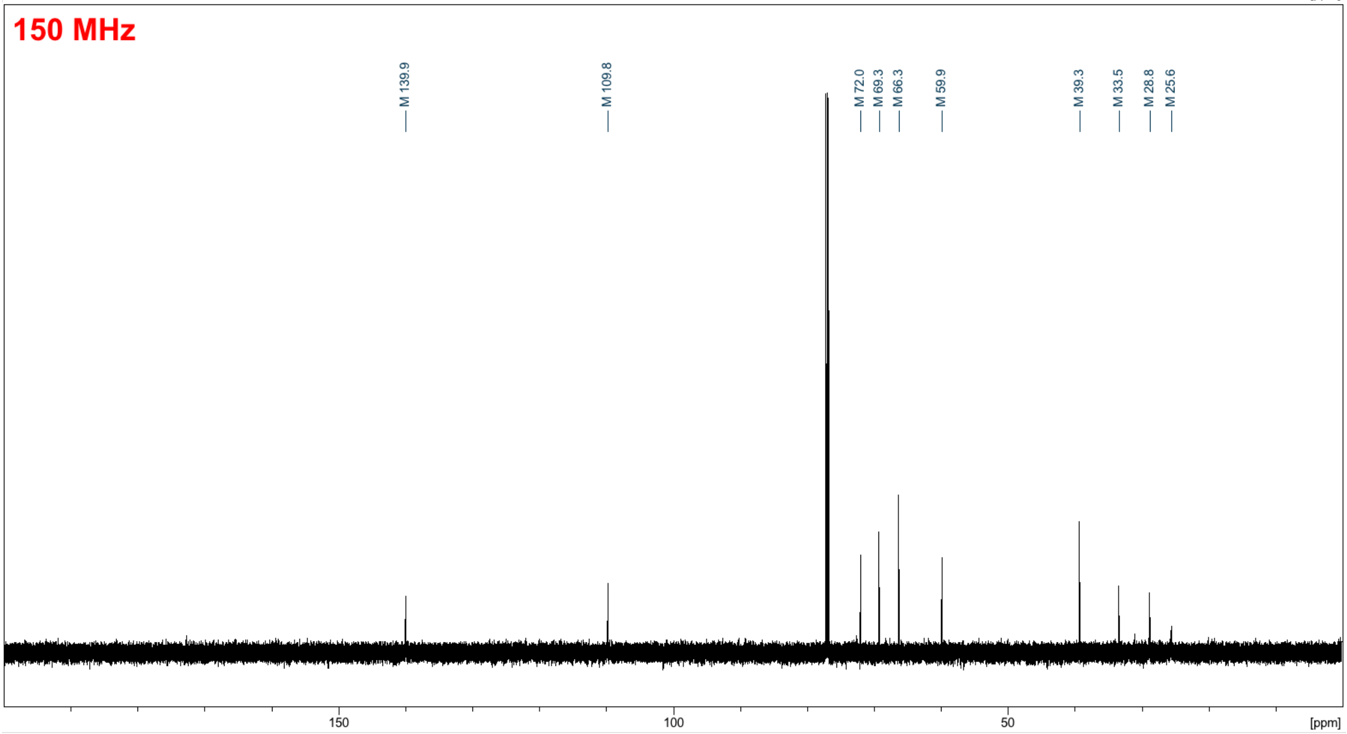
150 MHz
M 139.9
M 109.8
M 72.0
M 69.3
M 66.3
M 59.9
M 39.3
M 33.5
M 28.8
M 25.6
150
100
50
[ppm]

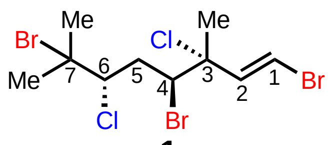

1

Distinct chlorine isotopic shift (Δδ = 9 ppb) observed at C3:

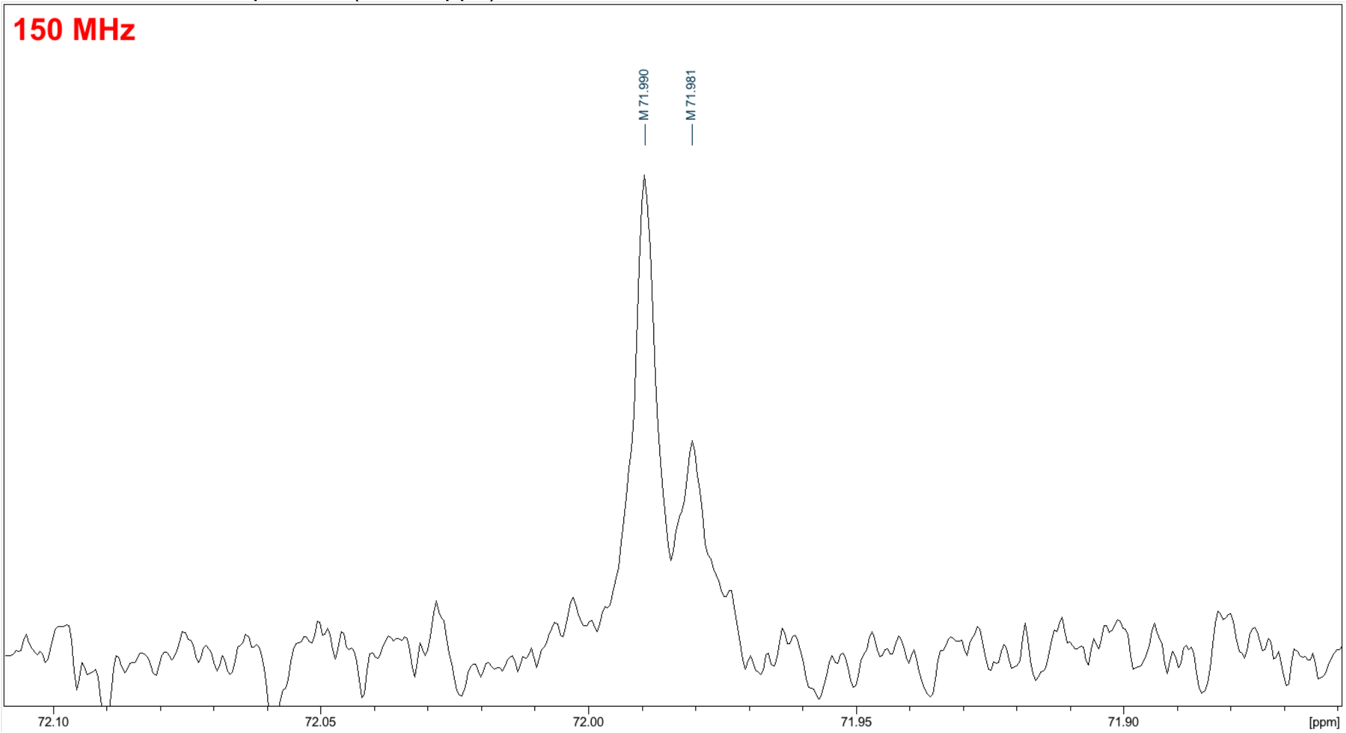

Chlorine isotopic shift (Δδ = 7 ppb) observed at C6, somewhat obscured by broadening:

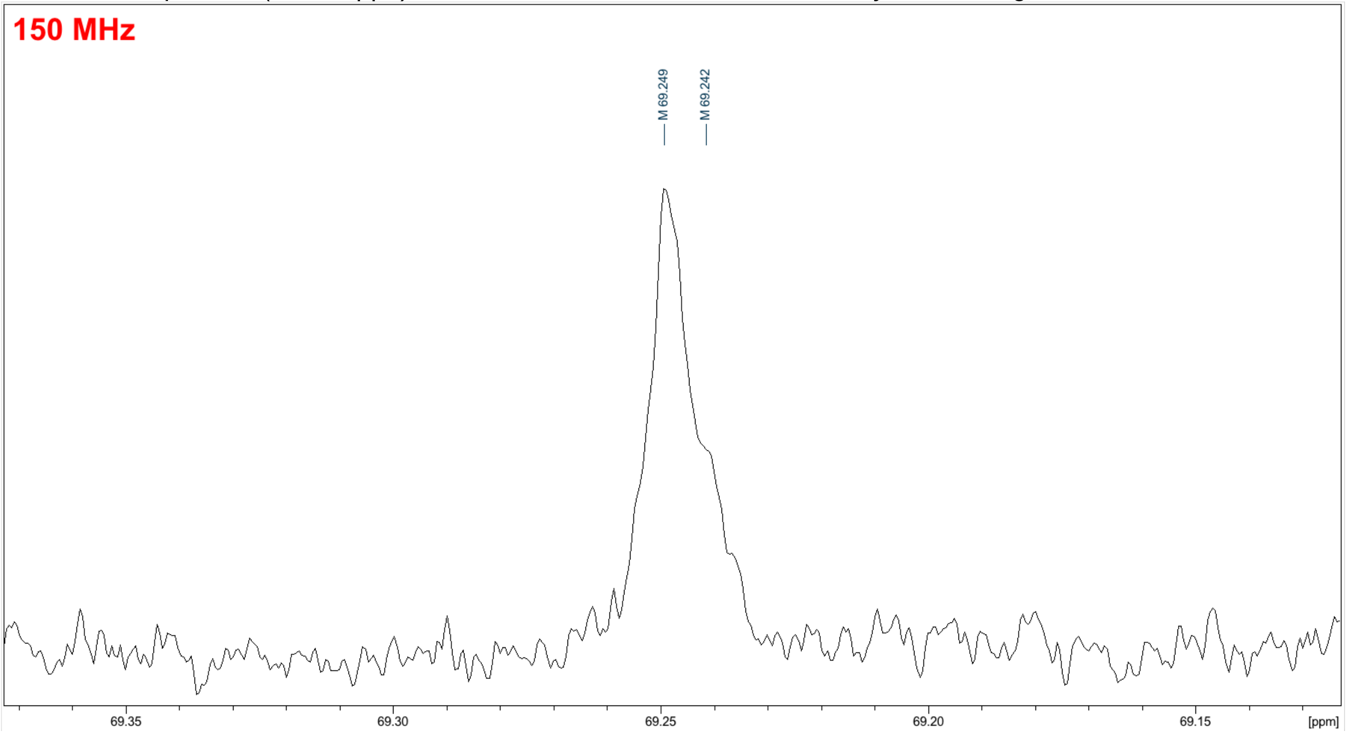

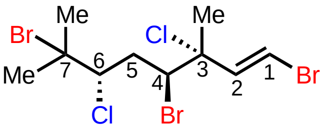

1

No chlorine isotopic shift seen at C4 (note broadening):

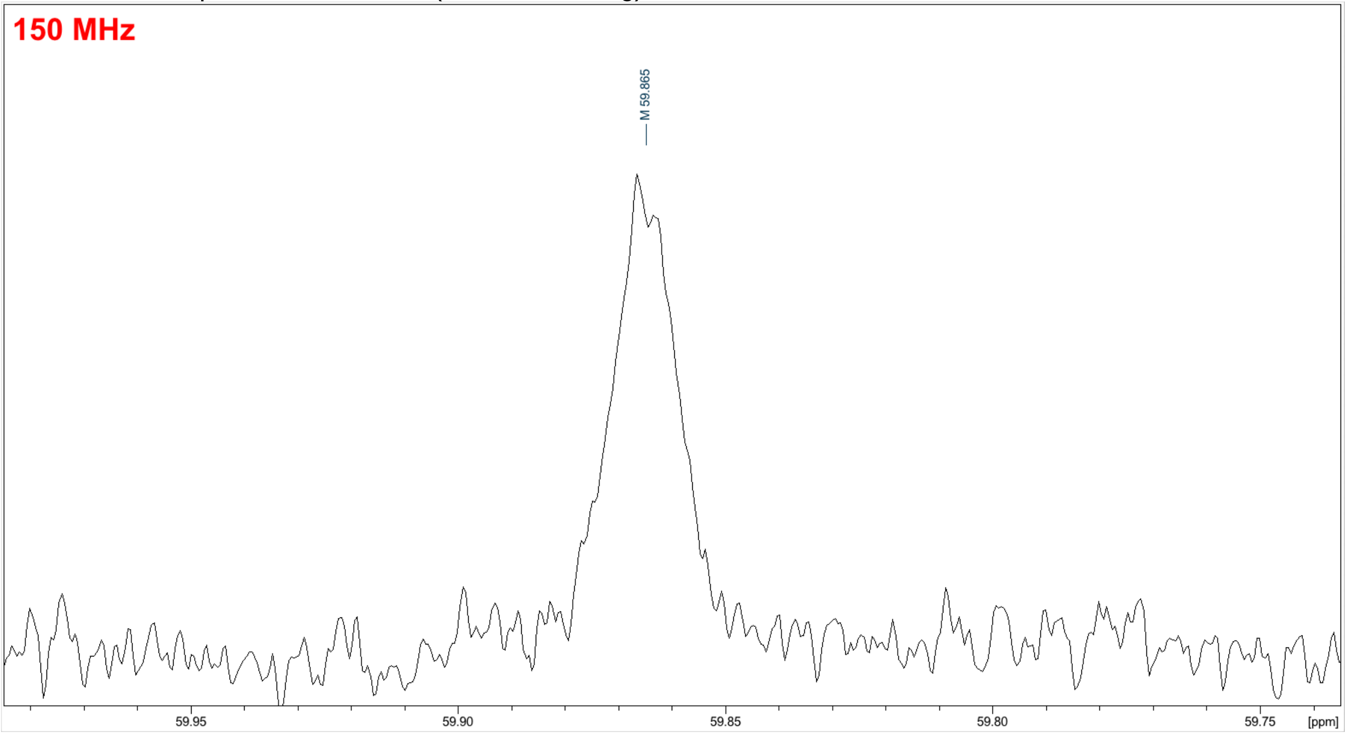

No chlorine isotopic shift seen at C6:

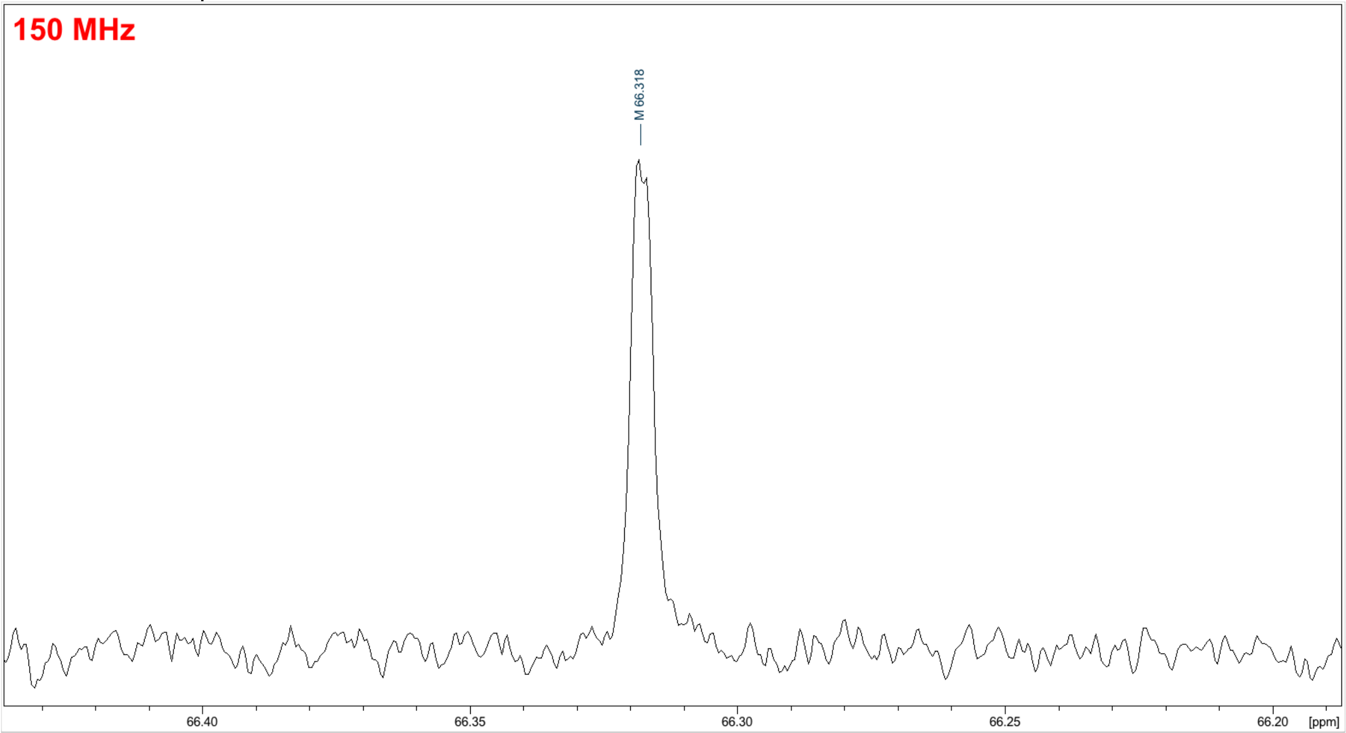

5. Comparison of synthetic and natural **1**:

Top: Natural **1** (Ankisetty et al. 2004) [4]; Bottom: Synthetic **1** [this work].

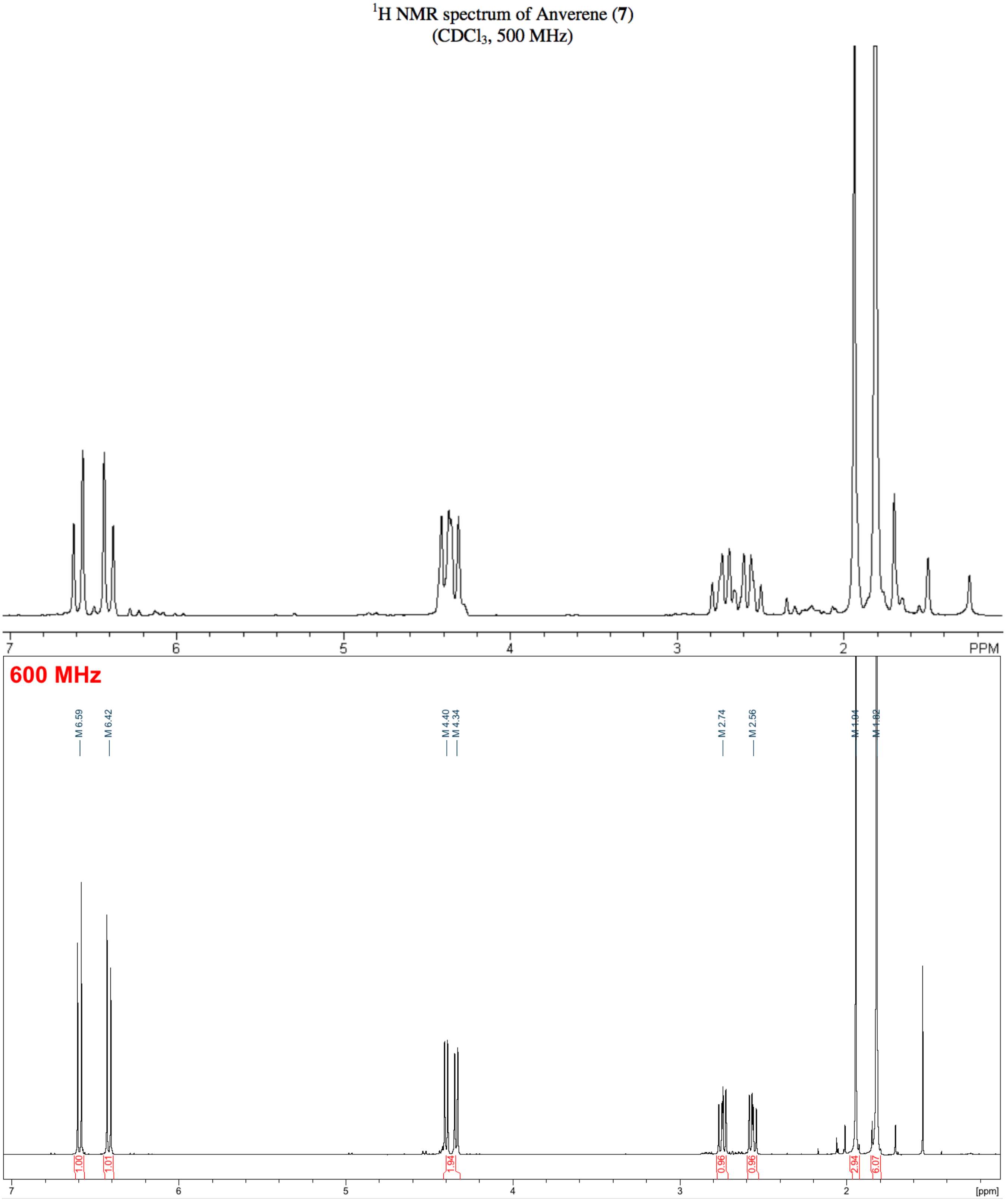

Table S2: Comparison of synthetic and natural (−)-anverene **1**.

^{1}H NMR data:

Synthetic **1** [this work] (600 MHz, $CDCl_3$)	Natural **1** (Ankisetty et al. 2004) [4] (500 MHz, $CDCl_3$)
6.59 (d, 1H, *J* = 13.5 Hz)	6.58 (d, 1H, *J* = 13.5)
6.42 (d, 1H, *J* = 13.5 Hz)	6.40 (d, 1H, *J* = 13.5)
4.40 (dd, 1H, *J* = 11.0, 1.6 Hz)	4.39 (dd, 1H, *J* = 10.7, 1.7 Hz)
4.34 (dd, 1H, *J* = 10.8, 1.5 Hz)	4.33 (dd, 1H, *J* = 10.7, 1.7 Hz)
2.74 (ddd, 1H, *J* = 14.9, 11.0, 1.6 Hz)	2.62 (m, 2H)
2.56 (ddd, 1H, *J* = 14.9, 10.8, 1.6 Hz)	
1.94 (s, 3H)	1.92 (s, 3H)
1.82 (overlapping s, 6H)	1.81 (s, 3H)
	1.81 (s, 3H)

^{13}C NMR data:

Synthetic **1** [this work] (150 MHz, $CDCl_3$)	Natural **1** (Ankisetty et al. 2004) [4] (62.5 MHz, $CDCl_3$)
139.9	139.9
109.8	109.7
72.0 (C–Cl)	71.9
69.3 (C–Cl)	69.2
66.3	66.3
59.9	59.8
39.3	39.2
33.5	33.4
28.8	28.8
25.6	25.5

6. HPLC traces of racemic and scalemic **6**:

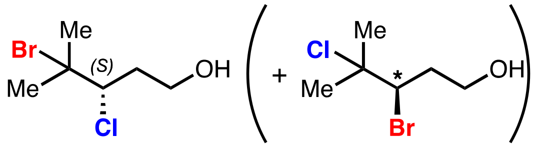

6 **7**

Racemic Sample (1:3 ratio of **6**:**7**; sample prepared by treatment of homoprenol **5** with PyHCl and NBS [1]): Chiralpak AD-H column, 5% EtOH in hexanes, 1 mL/min, 210 nm.

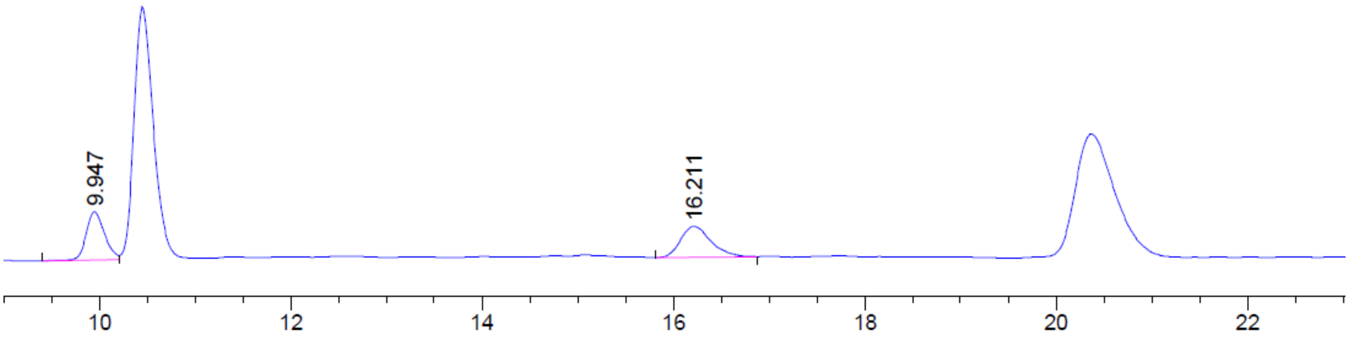

Peak #	RetTime [min]	Type	Width [min]	Area [mAU*s]	Height [mAU]	Area %
1	9.947	BV	0.2040	324.78421	24.29349	48.7481
2	16.211	BB	0.3374	341.46527	15.47637	51.2519
Totals :				666.24948	39.76986	

Scalemic Sample (88% ee for **6**, 8:1 ratio of **6**:**7**): Chiralpak AD-H column, 5% EtOH in hexanes, 1 mL/min, 210 nm.

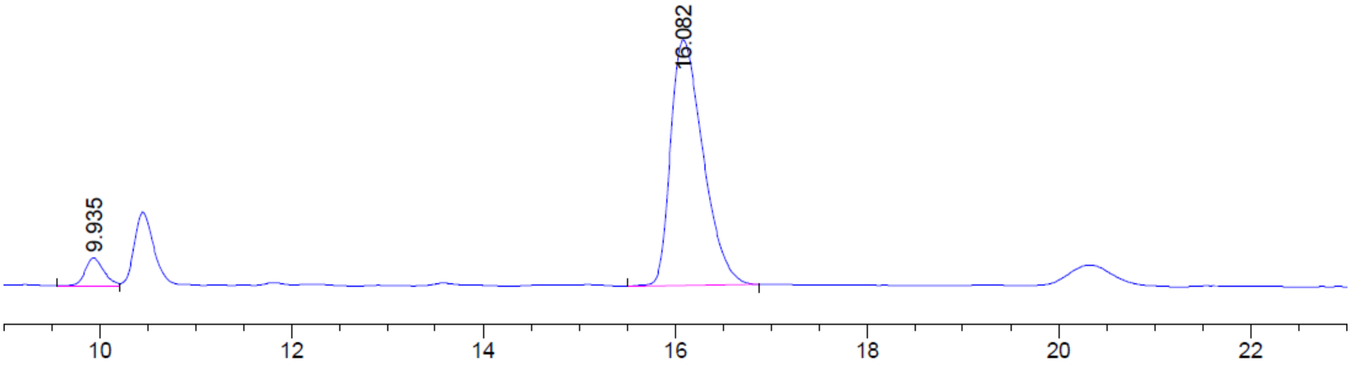

Peak #	RetTime [min]	Type	Width [min]	Area [mAU*s]	Height [mAU]	Area %
1	9.935	BV	0.2035	186.47798	13.81682	6.0797
2	16.082	BB	0.3642	2880.76367	122.56142	93.9203
Totals :				3067.24165	136.37825	

7. References

1. Hu, D. X.; Seidl, F. J.; Bucher, C.; Burns, N. Z. *J. Am. Chem. Soc.* **2015**, *137*, 3795–3798.

2. Hu, D. X. *Catalytic Enantioselective Dihalogenation of Allylic Alcohols and its Application to the Stereoselective Total Synthesis of Chlorosulfolipids*. Ph.D. Dissertation, Stanford University, Stanford, CA, **2015**.

3. Julia, M.; Julia, S.; Guégan, R. *Bull. Soc. Chim. Fr.* **1960**, 1072–1079.

4. Ankisetty, S.; Nandiraju, S.; Win, H.; Park, Y. C.; Amsler, C. D.; McClintock, J. B.; Baker, J. A.; Diyabalanage, T. K.; Pasaribu, A.; Singh, M. P.; Maiese, W. M.; Walsh, R. D.; Zaworotko, M. J.; Baker, B. J. *J. Nat. Prod.* **2004**, *67*, 1295–1302.

5. Aliev, A. E.; Harris, K. D. M. *Magn. Reson. Chem.* **1993**, *31*, 54−57.
